# Evaluation on Antidiabetic Properties of Medicinal Plants from Myanmar

**DOI:** 10.1155/2021/1424675

**Published:** 2021-08-26

**Authors:** Dongdong Zhang, Karuppusamy Arunachalam, Yuehu Wang, Yu Zhang, Jun Yang, Pyae Phyo Hein, Aye Mya Mon, Jianwen Li, Angkhana Inta, Xuefei Yang

**Affiliations:** ^1^Key Laboratory of Economic Plants and Biotechnology and the Yunnan Key Laboratory for Wild Plant Resources, Kunming Institute of Botany, Chinese Academy of Sciences, Kunming 650201, China; ^2^Southeast Asia Biodiversity Research Institute, Chinese Academy of Sciences, Yezin, Nay Pyi Taw 05282, Myanmar; ^3^Department of Biology, Faculty of Science, Chiang Mai University, 239 Huay Kaew Road, Chiang Mai 50200, Thailand

## Abstract

**Objectives:**

To explore the effective and safe medicines for treating diabetes.

**Methods:**

Hydroalcoholic extracts of 130 medicinal plants belonging to 66 families were evaluated using porcine pancreatic lipase (PPL) inhibition and glucose uptake methods together with a literature review.

**Results:**

The extracts of 22 species showed the PPL inhibition activity; 18 extracts of 15 species stimulated glucose uptake in 3T3-L1 adipocytes. Among them, *Mansonia gagei* J.R. Drumm., *Mesua ferrea* L., and *Centella asiatica* (L.) Urb. exhibited both activities. The extracts of *Caladium lindenii* (André) Madison rhizomes and *Azadirachta indica* A. Juss. leaves presented the utmost lipase inhibitory activity with IC_50_ of 6.86 ± 0.25 and 11.46 ± 0.06 *μ*g/mL, respectively. The extracts of *Coptis teeta* Wall. rhizomes and *Croton tiglium* L. seeds stimulated the maximum glucose uptake. Ten species are reported to have antidiabetic activity for the first time. Flavonoids and triterpenoids are the dominant antidiabetic compounds in selected medicinal plants from Myanmar.

**Conclusions:**

*P. zeylanica*, *L. cubeba, H. crenulate*, *M. gagei*, *C. teeta*, and *M. ferrea* are worthy to advance further study according to their strong antidiabetic activities and limited research on effects in *in vivo* animal studies, unclear chemical constitutes, and safety.

## 1. Introduction

Diabetes mellitus (DM) is a chronic metabolic disease and characterized by hyperglycemia that result from defects in insulin secretion, insulin action, or both [1]. Diabetes includes three main types, type I, type II, and gestational diabetes. Type II diabetes mellitus (T2DM) is the most common and accounts for over 90% of the total cases [2, 3]. T2DM has high morbidity and mortality worldwide and brings a large economic burden. Cardiovascular complications are a major cause of morbidity and mortality among people with diabetes [2]. The International Diabetes Federation reported that there were 463 million adults with diabetes in 2019, which have more than tripled over the past 20 years, and diabetes-related healthcare costs (USD 760 billion) are now estimated to account for 10% of the total healthcare budget (https://www.diabetesatlas.org/).

Most patients with T2DM are overweight or obese. Pancreatic lipase is an important enzyme for digestion and absorption of dietary lipids, and lipase inhibition is the most widely studied for the discovery of potential antiobesity agents [4]. Monogenic and common forms of obesity initially cause insulin resistance [5]. Insulin resistance stimulates glucose production in the liver and attenuates glucose uptake in muscle and adipose tissue which is one of the important factors resulting in T2DM [2]. As the behavior of 3T3-L1 adipocytes is similar to primary adipocytes and mostly available muscle cell lines are not insulin sensitive in glucose transport, 3T3-L1 adipocytes have become the most preferred cell line to study insulin-stimulated glucose uptake and an excellent cell model to study insulin action and signaling [6].

Currently, oral antidiabetic medications are mainly classified as biguanides, sulfonylureas, meglitinides, thiazolidinediones, dipeptidyl peptidase 4 (DPP-4) inhibitors, sodium-glucose cotransporter inhibitors, and *α*-glucosidase inhibitors [1]. These agents have adverse effects such as leading to hypoglycemia, cardiovascular, and metabolic alterations. People prefer medicinal plants for DM therapy due to low cost, minimal side effects, and inexpensive availability [1, 7]. In many parts of the world, traditional knowledge and biodiversity play an import role in health care, and many widely used plant-based medicines are derived from traditional knowledge [8]. However, only parts of medicinal plants have received scientific evaluation for their efficacy [7]. Thus, it is a challenge and an opportunity to search medicinal plants to treat diabetes and its complications.

Myanmar, a tropical country, possesses a rich resource of medicinal plants, which is widely practiced by the majority of the population [9]. However, there are limited pharmacological studies on traditional medicines. Aumeeruddy et al. reviewed the ethnomedicinal plants for the management of diabetes worldwide but lack of information in Myanmar [10]. To explore the pharmacological application of antidiabetic properties of traditional plants in Myanmar and search for new antidiabetic agents, we investigated three main regions of the medicinal plants source and reported their current research status on antidiabetes. These species were screened for the antidiabetic activities by porcine pancreatic lipase (PPL) inhibition and glucose uptake in 3T3-L1 adipocyte assays of selected medicinal plants in Myanmar.

## 2. Materials and Methods

### 2.1. Investigation and Collection of Medicinal Plant Materials

This study was carried out in the markets and villages of three regions including Mandalay, Sagaing, and Shan State in 2015–2017. These places possess affluent medicinal plants according to our survey in Myanmar. Mandalay has the biggest market of medicinal plants, which is Zay Cho market. Shan is the biggest state and the most famous for its traditional medicines in Myanmar [11]. The local name and usage of medicinal plants were recorded in the field survey. Meanwhile, the voucher specimens and materials were collected for identification and further research, respectively. Among them, 93 samples of medicinal plants from Mandalay were screened for antibacterial and anti-T3SS activities in our previous research [12]. The plants were identified by Ms. Jun Yang and Mr. Yu Zhang from the Kunming Institute of Botany based on specimens. The plant names and families were confirmed and conducted at the Plant List (http://www.theplantlist.org).

### 2.2. A Review of Medicinal Plants in Antidiabetic Research

To make clear the status quo on antidiabetes of these medicinal plants, we performed a thorough literature review by using electronic databases (Web of Science, PubMed, Google Scholar, and SciFinder). Specific keywords include “scientific name of species” and “diabetes” until 2021. The used parts of the species were searched about their antidiabetes including traditional use, *in vitro* assay, *in vivo* animal studies, clinical trials, and antidiabetic constitutes. Other parts were continued to search if the collected parts did not report any antidiabetic properties, and other species of the same genus were continued to collect information if the species did not report any antidiabetes. We used rigorous inclusion and exclusion criteria to select antidiabetic compounds with IC_50_ ≤ 50 *µ*M, or dose ≤50 *µ*M in *vitro*, or dose ≤50 mg/kg *in vivo*, excluding compounds with name or structure inconsistent with the source. Here, we refer to the antidiabetic activity of compounds with IC_50_ ≤ 10 *µ*M, dose ≤10 mg/kg in *vivo*, or dose ≤10 *µ*M *in vitro* as high and from 11 to 50 as moderate.

### 2.3. Preparation of Plant Extracts

The dried different parts of the plants were powdered in a grinder and then extracted with 70% ethanol at 60°C once for 30 minutes. After filtration, the solvent was removed under reduced pressure at 60°C in a rotary evaporator. The extracts were stored at 4°C before analysis.

### 2.4. Porcine Pancreatic Lipase (PPL) Inhibition Assay

Lipase inhibition activity was conducted as described in literature with some modifications [13]. Briefly, *p*-NPB was used as a substrate. The reactions were carried out in a 96-well microtiter plate where 5 *µ*L of lipase solution (40 U/mL) in Tris-HCl buffer (100 mM Tris-HCl, 5 mM CaCl_2_; pH 7.0) was used as enzyme buffer. Then, 1 *µ*L of each extract dissolved in dimethyl sulfoxide (DMSO) (20 mg/mL) and 184 *µ*L of the same Tris-HCl buffer were added and mixed with the enzyme buffer. After incubation at 37°C for 15 min, 10 *µ*L of the substrate solution (10 mM *p*-NPB in DMSO) was added. Then, enzymatic reactions were performed for 15 min at 37°C. Lipase activity was determined by measuring the hydrolysis of *p*-NPB to *p*-nitrophenol at 400 nm using a microplate reader, 630 nm as a reference. Orlistat was used as a positive control. The inhibitory activity (*I*) was calculated according to the following formula:

(1) $$\begin{array}{c} I\left(\%\right)=\left(1-\frac{A}{B}\right)\times 100\%. \end{array}$$

*A* is the optical density (OD) value of the sample. *B* is the OD value of negative control.

The half-maximal inhibitory concentration (IC_50_) was calculated by Reed and Muench's method.

### 2.5. Glucose Uptake in 3T3-L1 Adipocytes Assay

The differentiation of 3T3-L1 adipocytes and glucose uptake assay were carried out using a mild modified method described [14]. The differentiated 3T3-L1 adipocytes were seeded in 96-well plates and preincubated with the same way in reference. After that, they were incubated with 40 *μ*g/mL samples in medium. Insulin (0.1 *μ*mol/L) or berberine (10 *μ*mol/L) was used as positive control. DMSO was added as blank control. After 24 h, 10 *μ*L medium was taken to measure the glucose concentration by the glucose oxidase method. The experiments were repeated three times. Meanwhile, 20 *μ*L MTS was added in the remaining medium of cells and incubated 2 h at 37°C. Then, the absorbance at 492 nm was measured to determine the cytotoxicity of extracts. The value of glucose uptake of the sample was calculated using these formulas:

The glucose concentration (mmol/L) = sample absorbance/positive control absorbance × positive control concentration.

Glucose uptake (% of difference) = (glucose concentration of blank wells − remaining glucose in the cell-plated wells)/glucose concentration of blank wells ×100%.

## 3. Results

### 3.1. PPL Inhibition Activity of Plant Extracts

Twenty-two plant extracts (100 *μ*g/mL) showed the PPL inhibition activity (Table 1 and Table S1). Among them, eight species were tested, with the IC_50_ values according to PPL inhibition rate higher than 50% except for *Cuscuta* sp. in Table 1. *Cuscuta* sp. showed the PPL inhibition activity with an IC_50_ value of 93.52 ± 1.38 *μ*g/mL. *Cuscuta sp*. was not properly identified because of morphological characteristics without the flower or fruits. It was found in the same spot where *C. reflexa* was discovered. The ethanolic extracts of *C. lindenii* rhizomes and *A. indica* leaves possessed the highest lipase inhibitory activity with IC_50_ values of 6.86 ± 0.25 and 11.46 ± 0.06 *μ*g/mL, respectively. The ethanolic extracts of *P. zeylanica* stems, *G. glabra* roots, *C. pareira* aerial parts, *M. gagei* woods, and *C. reflexa* whole plants showed moderate lipase inhibitory activity with IC_50_ values of 37−95 *μ*g/mL. Fourteen species showed mild lipase inhibitory activity with 30–47% inhibition (Supplementary Materials, Table S1).

### 3.2. Glucose Uptake in 3T3-L1 Adipocytes of Plant Extracts

**Figure 1:**
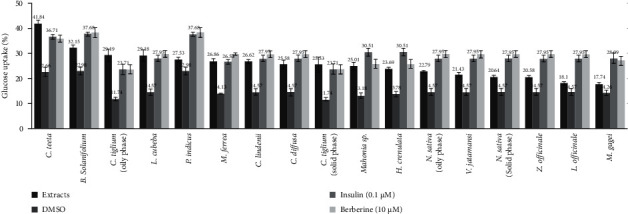
The effects 17 extracts (40 *μ*g/mL) of 15 plant species on glucose uptake of 3T3-L1 adipocytes. Data is represented as percentage of glucose uptake, in comparison to the control group (DMSO) and positive control (insulin and berberine).

Seventeen extracts (40 *μ*g/mL) and one plant extract reduced from 40 *μ*g/mL to 20 *μ*g/mL as cytotoxicity to cells of 15 species stimulated glucose uptake in 3T3-L1 adipocytes (Figure 1). The ethnobotanical information and pharmacological properties can be found in Table 2. *C. teeta* rhizomes and *C. tiglium* seeds (oily and solid phase) stimulated the highest increase in glucose uptake. At concentration 40 *μ*g/mL, their glucose uptakes were higher than positive controls which was insulin (0.1 *μ*M) and berberine (10 *μ*M). *Mahonia* sp. and *L. cubeba* fruits stimulated glucose uptake equivalent to berberine (10 *μ*M); *M. ferrea* leaves were close to insulin (0.1 *μ*M). The seed extracts of *C. tiglium* and *N. sativa* have oily and solid phase; the two phases were separated to screen the antidiabetic activities. Their oily phase of the extract showed a higher increase in glucose uptake. *Mahonia* sp. leaves significantly stimulated glucose uptake compared to the control group (Figure 1). The concentration was lowered to 20 *μ*g/mL for glucose uptake test as the root extracts of *H. crenulata* showed cytotoxicity against adipocytes at 40 *μ*g/mL. *H. crenulata* root significantly increased glucose uptake with the value of glucose uptake (21.81 ± 2.49) % at 20 *μ*g/mL compared to the control group (9.31 ± 1.34) %; the value of insulin (0.1 M) and berberine glucose uptake (10M) were (28.95 ± 1.16) % and (25.72 ± 1.45) %. This information was not mentioned in Figure 1.

### 3.3. Research Progress on Antidiabetic Activities of Medicinal Plants

To make clear the antidiabetic medicines for further study, the research status of medicinal plants in antidiabetes was reported in Supplementary Materials (Table S2) from traditional use to clinical research including traditional use, *in vitro* assay, *in vivo* animal studies, and clinical trials. The antidiabetic evidence of relative plants can be found in the Supplement Materials (Table S3).

**Figure 2:**
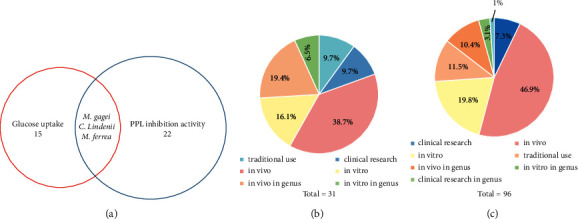
(a) The species possessed the PPL inhibition activity and stimulated glucose uptake in 3T3-L1 adipocytes. (b) Research status of traditional plants showing antidiabetic activities in our study. (c) Research status of extracted antidiabetic plants.

In our research, extracts (100 *μ*g/mL) of 22 species showed the PPL inhibition activity; 17 extracts (40 *μ*g/mL) and one plant extract reduced from 40 *μ*g/mL to 20 *μ*g/mL as cytotoxicity to cells of 15 species stimulated glucose uptake in 3T3-L1 adipocytes. Among them, *Caladium lindenii* (André) Madison, *Mansonia gagei* J.R. Drumm., and *Mesua ferrea* L. possessed both activities (Figure 2(a)). *Crateva religiosa* G. Forst., *Antidesma acidum* Retz., and *Coptis teeta* Wall. were the only reported traditional use for treating diabetes without any report on antidiabetic activity that accounted for 10% of research status (Figure 2(b)). Here, *Hesperethusa crenulata* (Roxb.) M. Roem., *M. gagei*, *A. acidum*, *Eriobotrya bengalensis* Kurz., *C. lindenii*, *Ligusticum officinale* (Makino) Kitag., *Cajanus volubilis* (King) Maesen, *Canscora diffusa* (Vahl) R.Br. ex Roem. and Schult., *Canscora andrographioides* Griff. ex C.B. Clarke, and *Pterocarpus indicus* Willd. were reported for their antidiabetic activities for the first time.

**Figure 3:**
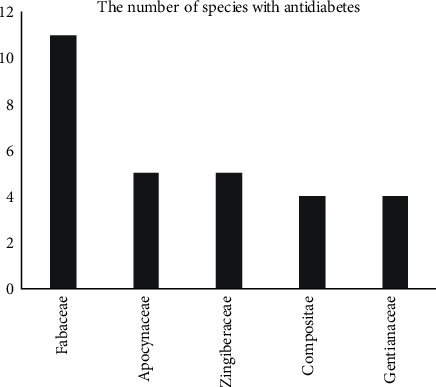
Top five plant families reported for antidiabetic properties by survey.

There are 82 species of 51 families and 14 species in the genus with antidiabetic properties so that they have high diversity (Supplementary Material, Tables S2 and S3). The Fabaceae family had the largest number of species showed antidiabetic activity (Figure 3). Among them, 7.3% species were already studied in type 2 diabetic patients, 46.9% species were researched antidiabetic activities *in vivo*, and 11.5% species were only recorded traditional use without any reported pharmacological activities on antidiabetes (Figure 2(c)).

### 3.4. A Literature Review of Antidiabetic Compounds from Selected Medicinal Plants in Myanmar

Forty of the 82 species were reported antidiabetic components with various types including terpenes, alkaloids, flavonoids, lignans, and other types (Figure S1). Among them, flavonoids (28 compounds) and triterpenoids (26 compounds) are the dominant compounds accounting for 61.4%. Flavonoids were mainly reported *α*-glucosidase inhibition and PPAR-*γ*-ligand-binding activities. They are found in *Oroxylum indicum*, *Eclipta prostrata*, *Nigella sativa*, *Glycyrrhiza glabra*, *Senna siamea*, *Boesenbergia rotunda*, and *Cinnamomum tamala*. PPAR-*γ*-ligand-binding activities in phenolic compounds are affected by the slight differences of substitution groups on the aromatic rings [33].

The primary targets of antidiabetic triterpenoids were *α*-glucosidase inhibition and lipolytic activity in 3T3-L1 adipocytes. Triterpenoids were isolated from *Abrus precatorius*, *Plumeria rubra*, *Pongamia pinnata*, *Entada phaseoloides*, *Eclipta prostrata*, *Lagerstroemia speciosa*, *Trigonella foenum-graecum*, *Centella asiatica*, *Brucea javanica*. Lupane skeleton and a ketone at C-3 of triterpenes would be essential for exerting a potent PPA inhibition in *A. precatorius* [34]. Triterpenoid glycosides having glucopyranosyl moiety are favorable for inhibiting PTP1B enzyme from *E. prostrata* [35]. Bitter-taste receptors of quassinoids may be a target as to induce lipolytic activity, the length of acyl side chain at 15-OH is important for the lipolytic activity of *B. javanica* [36].

## 4. Discussion

The potential species were discussed from pharmacological activities and safety extracts were with IC_50_ < 50 *μ*g/mL in PPL inhibition, higher or closely positive in glucose uptake, or showed these two activities.

*H. crenulata* is known as Thanaka, and the stem bark powder is used as a skincare regiment over one thousand years in Myanmar [31]. It is also a common tropical plant species in the Indian subcontinent and Southeast Asia with various medicinal properties, such as purgative, antidote, stomachic, and sudorific [31]. Coumarins and sitosterol were found in its root bark [37]. Extracts from Thanaka bark showed strong anti-inflammatory, significant antioxidation, and mild tyrosinase inhibition without detectable genotoxicity [31]. We reported the antidiabetic activities of its roots and barks for the first time.

The heartwood of *M. gagei* showed antibacterial, antifungal, and antioxidant activities [12, 17]. The chemical constitutes of *M. gagei* revealed antiestrogenic, antifungal, and antioxidant, and anticancer activities [17, 18]. Coumarins and mansonones are the main constituents and active ingredients in *M. gagei* [17, 18, 38, 39]. Its antidiabetic activities were reported for the first time which was proved with PPL inhibition and increase the glucose uptake to deserve further study about the activity *in vivo* and its safety.

The tuber of *C. lindenii* was used for Stingray wounds in Brazil [40]. The phytochemical investigation of *Caladium* indicated flavonoids, alkaloids, saponins, cardiac glycosides, carbohydrates, and deoxy sugars [41]. There is no reported research about the phytochemical and pharmacological research of *C. lindenii.* The corm of *C. bicolor* showed stimulation of glucose uptake in adipocytes and hepatoprotective activity in Hep G2 cells [42]. Methanol leaf extract of *C. bicolor* showed toxicity to the kidney but no adverse effect on the heart, lungs, spleen, liver, and brain [43].

*A. indica*, known as neem, is a common traditional medicine for the treatment with diabetes mellitus in Africa and India [44, 45]. At 25, 48.4, 93.5, 180.9, and 350 mg/kg body weight, intraperitoneal and oral administration of aqueous leaf extract of *A. indica* decreased the blood glucose levels in alloxan-induced diabetic mice but not in a dose-related manner [44]. Ten ligands of neem possessed binding properties with T2DM protein enzyme target phosphoenolpyruvate carboxykinase; compound 3-deacetyl-3-cinnamoyl-azadirachtin showed the best binding [46]. Meliacinolin possessed *α*-glucosidase and *α*-amylase inhibition activities with IC_50_ 46.7 and 32.2 *µ*g/mL from chloroform extract of *A. indica* leaves. And orally administered meliacinolin (20 mg/kg body weight) could be able to revert a set of biochemical parameters of streptozotocin STZ-diabetic mice to respective normal values and its mechanism was deduced via its insulinogenic action [45]. Braga et al. reviewed the safety of *A. indica* extracts and/or isolated compounds and revealed nontoxicity or less toxicity when orally administered but had acute toxicity by intramuscular injection or via the intraperitoneal route [47].

The roots and leaves of *P. zeylanica* are traditionally used to treat diabetes in India and China [48, 49]. The ethanol extracts of *P. zeylanica* stem inhibited porcine pancreatic lipase activity with IC_50_ 39.1 *μ*g/mL (Table 1). Oral administration of ethanolic extract of *P. zeylanica* roots (100 and 200 mg/kg) decreased blood and urine glucose levels, increased hepatic hexokinase activity, and decreased hepatic glucose-6-phosphatase, serum acid phosphatase, alkaline phosphatase, and lactate dehydrogenase in streptozotocin STZ-diabetic rats [50]. Plumbagin is the major bioactive component for antidiabetic activity (15 and 30 mg/kg body weight) by reducing blood glucose and returning other biochemical parameters to normal and enhancing GLUT4 mRNA, protein expression in diabetic rats from *P. zeylanica* root. The antidiabetic effect is lower than glibenclamide (4 mg/kg) [49]. Oral administration of aqueous extract was found to be safe up to the dose of 2000 mg/kg during the 14 days of observation by acute toxicity profile experiment [51].

The rhizome of *C. teeta* along with *C. chinensis* and *C. deltoidea* is known as “coptidis rhizome” which has been widely used to treat bacillary dysentery, diabetes, pertussis, sore throat, aphtha, and eczema in China [29]. *C. chinensis* polysaccharide (200 mg/kg body weight) showed significant inhibition in fasting blood glucose level and triglycerides [52]. Alkaloids are the most abundant components and are considered as the main active ingredients [29]. The methanol extract of *C. chinensis* rhizome significantly inhibits adipocyte differentiation and lipid contents in 3T3-L1 cells. Five alkaloids, berberine, epiberberine, coptisine, palmatine, and magnoflorine were significantly inhibited lipid accumulation in 3T3-L1 cells without affecting cell viability and reduced the expression levels of several adipocyte marker genes including proliferator-activated receptor-*γ* (PPAR-*γ*) and CCAAT/enhancer-binding protein-*α* (C/EBP-*α*) from methanol extract of *C. chinensis* rhizome [53]. (3*β*)-Stigmast-5-en-3-ol stimulates glucose uptake by the PI3K-dependent pathway in L6 myotubes and activates GLUT 4 transport [54, 55]. Berberine was the main compound from the ethanol extract of *C. teeta* rhizome [54]. Lan et al. extracted 27 clinical trials (2569 patients) and revealed berberine with comparable therapeutic effects on T2DM, hyperlipidemia, and hypertension with no serious adverse reactions [56].

*C. tiglium* seeds were traditionally used for stimulating appetite, imbalances in phlegm and gas, jaundice, fainting, and facial paralysis and taken as a laxative to rid the body of impurities in Myanmar. The seeds oil can be used for stomach disorders, hypertension, fever, inflammation, infections, and diseases of the throat and ear [8]. *C. cajucara*, *C. macrostachys*, and *C. malambo* were used to cure diabetes [57]. The extract of *C. tiglium* inhibited *α*-amylase with 55.1% at 80 *μ*g/mL [16]. Oral medication can cause severe gastrointestinal syndrome and even mortality because of irritating oils and croton proteins [58]. Tigliane-type diterpenoids are the predominant secondary metabolite constituents in *C. tiglium* L. [59].

The raw fruits of *L. cubeba* were traditionally used to prevent and check for hyperglycemia in India. Its methanol extract inhibited *α*-amylase and *α*-glucosidase with IC_50_ values of 514.9 *µ*g/mL and 1,435.7 *µ*g/mL, respectively. Its ethanol extracts exhibited lower inhibition activity. Phenols and flavonoids were the major phytochemicals in different extracts against diabetes [60]. *L. cubeba* oil was proved with slightly toxic that the oral LD_50_, dermal LD_50_, and inhalation LC_50_ values were approximately 4,000 mg/kg of body weight [61].

The buds of *M. ferrea* displayed the mild inhibition on *α*-glucosidase and *α*-amylase activity with IC_50_ values of 128.8 *µ*g/mL and 146.8 *µ*g/mL, respectively [62, 63]. Stamens of *M. ferrea* produced a dose-dependent reduction in blood sugar (hypoglycemia) at a dose of 150 and 300 mg/kg body weight in alloxan-induced diabetic rats [64]. The methanol extract of *M. ferrea* flower has shown the antidiabetic activity in streptozotocin-induced diabetic rats and deduced the mechanism [65]. The extract of *M. ferrea* flower was proved no acute toxicity in an animal model but exhibited mild lymphocytic infiltration and hepatocyte degeneration [65, 66]. Xanthones and coumarins are predominantly secondary metabolites from *Mesua* species which display antitumor and antimicrobial, antioxidant, anti-inflammatory, or immunomodulating properties [67]. However, there is not any research reported related with antidiabetic activity of its leaves previously.

## 5. Conclusion

*L. cubeba, H. crenulate*, and *M. gagei* deserve further study because of the potential antidiabetic activity in *in vitro* assay and unclear active ingredients and mechanism along with research about antidiabetic effects in *in vivo* animal studies and unclarity in safety except for *L. cubeba* with slightly toxic activity. Moreover, *C. teeta*, *P. zeylanica*, and *M. ferrea* are also worth giving attention as the low toxicity and antidiabetic activities of these species or its genus level in research status. The antidiabetic activities and their constitutes of *M. ferrea* are studied in our research.

## Figures and Tables

**Table 1:** Ethnobotanical information and the PPL inhibition activity of seven species.

Scientific name	Family	Myanmar name	Common name	Part^a^	Yield^b^ (%)	Traditional use in Myanmar	IC_50_ (*μ*g/mL)
*Azadirachta indica* A. Juss.	Meliaceae	Tama	Neem	L	12	The decoction of leaves is orally taken for diabetes and antiseptic purpose. Leaves are externally used for skin diseases [9]	11.46 ± 0.06
*Caladium lindenii* (André) madison	Araceae	Gamon-gya	Indian kale	Rh	21	Undocumented	6.86 ± 0.25
*Plumbago zeylanica* L.	Plumbaginaceae	Kant-gyoke-phyu	White leadwort	St	11	Whole plants are used for stimulating palate and digestion and treating diarrhea, gastric diseases, and herpes-like skin disorders [8]	39.06 ± 1.11
*Glycyrrhiza glabra* L.	Leguminosae	Thinbaw-nwecho	Liquorice	R	20	Undocumented	58.36 ± 6.58
*Cuscuta reflexa Roxb.*	Convolvulaceae	Shwe-nwe	Dodder	WP	13	Whole plants are used to treat irregularities of the blood. The boiling liquid of whole plants is used for inflammation and hardening of the liver. Whole plants are crushed and pasted for itches and rashes [8].	78.83 ± 3.68
*Cissampelos pareira L*.	Menispermaceae	Kywet-nabaung	Velvet leaf	AP	20	Whole plants are pasted for inflammatory conditions of the eye. Leaves are used for cooling [8].	87.71 ± 3.01
*Mansonia gagei* J. R. Drumm.	Malvaceae	Kala-met	Bustard sandalwood	W	4	Woods and roots are used to eliminate phlegm, to treat heart diseases, urinary disorders, and anemia, and to alleviate itches [8]	88.91 ± 0.96

^a^L: leaves, AP: aerial parts, W: woods, St: stems, Rh: rhizomes, R: roots, and WP: whole plants. ^b^Yield (%) = (weight of extract/weight of dry sample)×100.

**Table 2:** Ethnobotanical information and pharmacological properties of species with stimulating glucose uptake.

Scientific name	Myanmar name	Family	Part^a^	Yield (%)	Traditional use in Myanmar	Pharmacological properties
*Croton tiglium L*.	Kanakho, mai-hkang	Euphorbiaceae	S (oily phase)	9	Oil can be used for stomach disorders, hypertension, fever, inflammation, infections, and diseases of the throat and ear [8]	Antioxidant, antitumor, anti-HIV, anticonvulsant, gastrointestinal, anti-inflammatory, and *α*-amylase inhibitory activities [15, 16]
*Croton tiglium L*.	Kanakho, mai-hkang	Euphorbiaceae	S (solid phase)	7	To stimulate appetite; correct imbalances in phlegm and gas; prevent jaundice, fainting, and facial paralysis; also taken as a laxative to rid the body of impurities [8]	Antioxidant, antitumor, anti-HIV, anticonvulsant, gastrointestinal, anti-inflammatory, and *α*-amylase inhibitory activities [15, 16]
*Mansonia gagei* J.R. Drumm.	Kala-met	Malvaceae	W	4	To eliminate phlegm; to treat heart diseases, urinary disorders, and anemia; to alleviate itches [8]	Anticancer, antifungal, antioxidant, and larvicidal activities [17, 18]
*Litsea cubeba* (lour.) pers.	Thing ting	Lauraceae	Fr	21	To treat stomachache and stomach ulcer; used for postpartum care [19]	Antimicrobial, anti-inflammatory, antiasthmatic, anticholinergic, and antiplatelet aggregation activities [20]
*Valeriana jatamansi* jones	Kanpalu	Caprifoliaceae	Rh	22	Treating for diabetes	Analgesic, anti-inflammatory, myorelaxant and antispasmodic, psychotic, radioprotective, antimicrobial, hypnotic, anthelmintic, cytotoxic, and antioxidant activities [21]
*Ligusticum officinale* (makino) kitag.	Kantbalu-u-thae	Apiaceae	Rh	12	Undocumented	Pancreatic lipase inhibitory, anti-inflammatory, anticancer, and antioxidant activities [22]
*Canscora diffusa* (vahl) R.Br. ex roem. and schult.	Kyauk-pan	Gentianaceae	L	19	Undocumented	Antibacterial activities [23]
*Caladium lindenii* (andré) madison	Gamone-gya	Araceae	Rh	21	Undocumented	Not reported
*Zingiber officinale* roscoe	Gyin	Zingiberaceae	Rh	11	Laryngitis, chest and respiratory ailments, infected sores, and inflammation caused by injury [8]	Antidiabetic, antithrombotic, anti-inflammatory, analgesic, antioxidant, and antimicrobial activities [24]
*Nigella sativa L*.	Samon-net	Ranunculaceae	S (oily phase)	3	Carminative and galactagogue [8]	Antimicrobial, antioxidant, antihyperlipidemic, anticancer, antidiabetic, gastroprotective, nephroprotective, and hepatoprotective activities [25]
*Nigella sativa L*.	Samon-net	Ranunculaceae	S (solid phase)	12	Used as a carminative and galactagogue [8]	Antimicrobial, antioxidant, anti-inflammatory, antihyperlipidemic, anticancer, antidiabetic, cardiovascular protective, gastroprotective, nephroprotective, hepatoprotective, wound-healing activities, the effect on the reproductive system, and immunoprotective activity [25]
*Baliospermum solanifolium* (Burm.) Suresh	Hnat-cho, ya-wo-mo, ya-wo-po	Euphorbiaceae	St	4	Toothache, purge	Roots have anticancer, immunomodulatory, anti-inflammatory, antioxidant, and hepatoprotective activities [26]
*Pterocarpus indicus* Willd.	Pan-padauk	Leguminosae	W	5	Dysentery, diarrhea	Barks showed *α*-glucosidase inhibitory activity [27], antitumor and antibacterial activity, and antiallergic properties [28]
*Coptis teeta* Wall.	Khan tauk	Ranunculaceae	R	23	To relieve constipation, regulate bowel movements, promote digestion, reduce fever, treat malaria, and increase vitality; roots soaked in liquor for malaria [8]	Antipathogenic microorganism, antidiabetes, and anticancer activities; protective effects on the cardiovascular system [29]
*Mesua ferrea L*.	Guntgaw	Calophyllaceae	L	12	To treat snakebites [8]	Analgesic and antibacterial activities [30]
*Hesperethusa crenulata* (Roxb.) Roem.	Sansph-ka, Thanaka	Rutaceae	R	9	Barks are used as a skincare [31].	Barks showed antioxidant, anti-inflammatory, and tyrosinase inhibitory activities [31]; leaves showed tyrosinase inhibitory activity [32]
*Hesperethusa crenulata* (Roxb.). Roem.	Sansph-ka, Thanaka	Rutaceae	L	5	Barks are used as a skincare [31].	Barks showed antioxidant, anti-inflammatory, and tyrosinase inhibitory activities [31]; leaves showed tyrosinase inhibitory activity [32]

^a^S: seeds, L: leaves, R: roots, Rh: rhizomes, W: woods, WP: whole plants, St: stems, and Fr: fruits.

## Data Availability

The data used to support the findings of this study are included within the Supplementary Materials.

## Supplementary Materials

Supplementary Materials *Table S1*. Information with 30–50% PPL inhibition activities of 17 medicinal plants. *Table S2*. 82 species of 51 families with antidiabetic properties research status. *Table S3*. Relative plants in antidiabetic reports without any antidiabetes research. *Table S4.* The information of antidiabetic ingredients. *Figure S1*. Antidiabetic components from medicinal plants in Myanmar.

## References

[1] Chaudhury A., Duvoor C., Reddy Dendi V. S. (2017). Clinical review of antidiabetic drugs: implications for type 2 diabetes mellitus management. *Frontiers in Endocrinology*.

[2] Zheng Y., Ley S. H., Hu F. B. (2018). Global aetiology and epidemiology of type 2 diabetes mellitus and its complications. *Nature Reviews Endocrinology*.

[3] Ali Asgar M. (2012). Anti-diabetic potential of phenolic compounds: a review. *International Journal of Food Properties*.

[4] Lunagariya N. A., Patel N. K., Jagtap S. C., Bhutani K. K. (2014). Inhibitors of pancreatic lipase: state of the art and clinical perspectives. *EXCLI journal*.

[5] Czech M. P. (2017). Insulin action and resistance in obesity and type 2 diabetes. *Nature Medicine*.

[6] Lakshmanan J., Elmendorf J. S., Özcan S. (2003). *Analysis of Insulin-Stimulated Glucose Uptake in Differentiated 3T3-L1 Adipocytes*.

[7] Manukumar H. M., Shiva Kumar J., Chandrasekhar B., Raghava S., Umesha S. (2017). Evidences for diabetes and insulin mimetic activity of medicinal plants: present status and future prospects. *Critical Reviews in Food Science and Nutrition*.

[8] DeFilipps R. A., Krupnick G. A. (2018). The medicinal plants of Myanmar. *PhytoKeys*.

[9] (2007).

[10] Aumeeruddy M. Z., Mahomoodally M. F. (2021). Ethnomedicinal plants for the management of diabetes worldwide: a systematic review. *Current Medicinal Chemistry*.

[11] Tran Q. L., Tran Q. K., Kouda K. (2003). Investigation on traditional medicine in Myanmar and Vietnam. *Journal of Traditional Medicines*.

[12] Li T. H., Zhang D. D., Oo T. N. (2018). Investigation on the antibacterial and anti-T3SS activity of traditional Myanmar medicinal plants. Evidence-based complementary and alternative medicine. *Evidence-Based Complementary and Alternative Medicine*.

[13] Kim J. H., Kim H. J., Park H. W., Youn S. H., Choi D.-Y., Shin C. S. (2007). Development of inhibitors against lipase and Î±-glucosidase from derivatives of monascus pigment. *FEMS Microbiology Letters*.

[14] Zhou L., Yang Y., Wang X. (2007). Berberine stimulates glucose transport through a mechanism distinct from insulin. *Metabolism*.

[15] Sinsinwar S., Paramasivam I., Muthuraman M. S. (2016). An overview of the biological and chemical perspectives of *Croton tiglium*. *Scholars Research Library*.

[16] Karthik V. P., Punnagai P., Suresh P. (2019). In Vitro hydrogen peroxide scavenging activity and alpha amylase inhibitory activity of *Croton tiglium* extract. *Research Journal of Pharmacy and Technology*.

[17] Tiew P., Ioset J.-R., Kokpol U., Chavasiri W., Hostettmann K. (2003). Antifungal, antioxidant and larvicidal activities of compounds isolated from the heartwood of *Mansonia gagei*. *Phytotherapy Research*.

[18] Baghdadi M. A., Al-Abbasi F. A., El-Halawany A. M., Aseeri A. H., Al-Abd A. M. (2018). Anticancer profiling for coumarins and related O-naphthoquinones from *Mansonia gagei* against solid tumor cells in vitro. *Molecules*.

[19] Ong H. G., Ling S. M., Win T. T. M., Kang D.-H., Lee J.-H., Kim Y.-D. (2018). Ethnobotany of wild medicinal plants used by the Müün ethnic people: a quantitative survey in southern Chin state, Myanmar. *Journal of Herbal Medicine*.

[20] Kong D.-G., Zhao Y., Li G.-H. (2015). The genus *Litsea* in traditional Chinese medicine: an ethnomedical, phytochemical and pharmacological review. *Journal of Ethnopharmacology*.

[21] Devi V. S., Rao M. G. (2014). *Valeriana wallichii*–a rich aroma root plant. *World Journal of Pharmacy and Pharmaceutical Sciences*.

[22] Mo E. J., Yang H. J., Jeong J. Y. (2016). Pancreatic lipase inhibitory phthalide derivatives from the rhizome of *Cnidium officinale*. *Records of Natural Products*.

[23] Mahida Y., Mohan J. S. S. (2008). Screening of Indian plant extracts for antibacterial activity. *Pharmaceutical Biology*.

[24] Ali B. H., Blunden G., Tanira M. O., Nemmar A. (2008). Some phytochemical, pharmacological and toxicological properties of ginger (*Zingiber officinale* Roscoe): a review of recent research. *Food and Chemical Toxicology*.

[25] Wesam K., Zahra H. N., Naim S. A., Majid A. S., Damoon A. L. (2016). Phytochemistry, pharmacology, and therapeutic uses of black seed (*Nigella sativa*). *Chinese Journal of Natural Medicines*.

[26] Mali R., Wadekar R. (2008). Baliospermum montanum (Danti): ethnobotany, phytochemistry and pharmacology- A review. *International Journal of Green Pharmacy*.

[27] Sichaem J., Tip-pyang S., Lugsanangarm K., Jutakanoke R. (2018). Highly potent *α*-glucosidase inhibitors from Pterocarpus indicus and molecular docking studies. *Songklanakarin Journal of Science and Technology*.

[28] Cha H.-S., Kim W.-J., Lee M.-H. (2016). Inhibitory effect of *Pterocarpus indicus* Willd water extract on IgE/Ag-induced mast cell and atopic dermatitis-like mouse models. *Bioscience, Biotechnology, and Biochemistry*.

[29] Wang J., Wang L., Lou G.-H. (2019). Coptidis Rhizoma: a comprehensive review of its traditional uses, botany, phytochemistry, pharmacology and toxicology. *Pharmaceutical Biology*.

[30] Chahar K., Mesua ferrea L. (2013). Mesua ferrea L.: a review of the medical evidence for its phytochemistry and pharmacological actions. *African Journal of Pharmacy and Pharmacology*.

[31] Wangthong S., Palaga T., Rengpipat S., Wanichwecharungruang S. P., Chanchaisak P., Heinrich M. (2010). Biological activities and safety of Thanaka (*Hesperethusa crenulata*) stem bark. *Journal of Ethnopharmacology*.

[32] Myint K. Z. W.. Phytochemical and in Vitro Biological Studies on Myanmar Medicinal Plants for Functional Food and Cosmetic Applications.

[33] Kuroda M., Mimaki Y., Honda S., Tanaka H., Yokota S., Mae T. (2010). Phenolics from Glycyrrhiza glabra roots and their PPAR-*γ* ligand-binding activity. *Bioorganic & Medicinal Chemistry*.

[34] Yonemoto R., Shimada M., Gunawan-Puteri M. D. P. T., Kato E., Kawabata J. (2014). *α*-amylase inhibitory triterpene from *Abrus precatorius* leaves. *Journal of Agricultural and Food Chemistry*.

[35] Le D. D., Nguyen D. H., Ma E. S. (2021). PTP1B inhibitory and anti-inflammatory properties of constituents from *Eclipta prostrata* L. *Biological and Pharmaceutical Bulletin*.

[36] Lahrita L., Moriai K., Iwata R., Itoh K., Kato E. (2019). Quassinoids in *Brucea javanica* are potent stimulators of lipolysis in adipocytes. *Fitoterapia*.

[37] Nayar M. N. S., Bhan M. K. (1972). Coumarins and other constituents of *Hesperethusa crenulata*. *Phytochemistry*.

[38] Tiew P., Puntumchai A., Kokpol U., Chavasiri W. (2002). Coumarins from the heartwoods of *Mansonia gagei* drumm. *Phytochemistry*.

[39] Mongkol R., Chavasiri W. (2016). Antimicrobial, herbicidal and antifeedant activities of mansonone E from the heartwoods of Mansonia gagei Drumm. *Journal of Integrative Agriculture*.

[40] Coelho-Ferreira M. (2009). Medicinal knowledge and plant utilization in an Amazonian coastal community of Marudá, Pará State (Brazil). *Journal of Ethnopharmacology*.

[41] Essien E. E., Jacob I. E., Thomas P. S. (2015). Phytochemical composition, antimicrobial and antioxidant activities of leaves and tubers of three *Caladium* species. *International Journal of Medicinal Plants and Natural Products*.

[42] Abima Shazhni J. R., Renu A., Vijayaraghavan P. (2018). Insights of antidiabetic, anti-inflammatory and hepatoprotective properties of antimicrobial secondary metabolites of corm extract from *Caladium x hortulanum*. *Saudi Journal of Biological Sciences*.

[43] Akhigbemen A. M., Ozolua R. I., Bafor E. E., Okwuofu E. O. (2018). Subacute toxicological profile of *Caladium bicolor* Aiton (Araceae) methanolic leaf extract in rat. *Journal of Pharmacy & Pharmacognosy Research*.

[44] Arika W. M., Nyamai D. W., Agyirifo D. S., Ngugi M. P., Njagi E. N. M. (2016). In vivo antidiabetic effect of aqueous leaf extract of *Azardirachta indica* A. juss in alloxan induced diabetic mice. *Journal of Diabetic Complications and Medicine*.

[45] Perez-Gutierrez R. M., Damian-Guzman M. (2012). Meliacinolin: a potent *α*-glucosidase and *α*-amylase inhibitor isolated from Azadirachta indica leaves and in vivo antidiabetic property in streptozotocin-nicotinamide-induced type 2 diabetes in mice. *Biological & Pharmaceutical Bulletin*.

[46] Jalil A., Ashfaq U. A., Ashfaq U. A. (2013). Screening and design of anti-diabetic compounds sourced from the leaves of neem (*Azadirachta indica*). *Bioinformation*.

[47] Braga T. M., Rocha L., Chung T. Y. (2021). *Azadirachta indica* A. Juss. In vivo toxicity-an updated review. *Molecules*.

[48] Goyal M. (2015). Traditional plants used for the treatment of diabetes mellitus in Sursagar constituency, Jodhpur, Rajasthan - an ethnomedicinal survey. *Journal of Ethnopharmacology*.

[49] Sunil C., Duraipandiyan V., Agastian P., Ignacimuthu S. (2012). Antidiabetic effect of plumbagin isolated from *Plumbago zeylanica* L. root and its effect on GLUT4 translocation in streptozotocin-induced diabetic rats. *Food and Chemical Toxicology*.

[50] Zarmouh M. M., Subramaniyam K., Viswanathan S., Kumar P. G. (2010). Cause and effect of *Plumbago zeylanica* root extract on blood glucose and hepatic enzymes in experimental diabetic rats. *African Journal of Microbiology Research*.

[51] Pendurkar S. R., Mengi S. A. (2009). Antihyperlipidemic effect of aqueous extract ofPlumbago zeylanicaroots in diet-induced hyperlipidemic rat. *Pharmaceutical Biology*.

[52] Jiang S., Wang Y., Ren D. (2015). Antidiabetic mechanism ofCoptis chinensispolysaccharide through its antioxidant property involving the JNK pathway. *Pharmaceutical Biology*.

[53] Choi J. S., Kim J.-H., Ali M. Y., Min B.-S., Kim G.-D., Jung H. A. (2014). Coptis chinensis alkaloids exert anti-adipogenic activity on 3T3-L1 adipocytes by downregulating C/EBP-*α* and PPAR-*γ*. *Fitoterapia*.

[54] Payum T. (2017). Distribution, ethnobotany, pharmacognosy and phytoconstituents of *Coptis teeta* Wall.: a highly valued and threatened medicinal plant of eastern himalayas. *Pharmacognosy Journal*.

[55] Sujatha S., Anand S., Sangeetha K. N. (2010). Biological evaluation of (3*β*)-STIGMAST-5-EN-3-OL as potent anti-diabetic agent in regulating glucose transport using in vitro model. *International Journal of Diabetes Mellitus*.

[56] Lan J., Zhao Y., Dong F. (2015). Meta-analysis of the effect and safety of berberine in the treatment of type 2 diabetes mellitus, hyperlipemia and hypertension. *Journal of Ethnopharmacology*.

[57] Salatino A., Salatino M. L. F., Negri G. (2007). Traditional uses, chemistry and pharmacology of Croton species (euphorbiaceae). *Journal of the Brazilian Chemical Society*.

[58] Liu L., Yu H., Wu H. (2017). Toxic proteins from *Croton tiglium* L. exert a proinflammatory effect by inducing release of proinflammatory cytokines and activating the p38-MAPK signaling pathway. *Molecular Medicine Reports*.

[59] Xu W. H., Liu W. Y., Liang Q. (2018). Chemical constituents from Croton species and their biological activities. *Molecules*.

[60] Chakraborty R., Mandal V. (2018). In vitro hypoglycemic and antioxidant activities of *Litsea cubeba* (lour.) pers. Fruits, traditionally used to cure diabetes in darjeeling hills (India). *Pharmacognosy Journal*.

[61] Luo M., Jiang L.-K., Zou G.-L. (2005). Acute and genetic toxicity of essential oil extracted from *Litsea cubeba* (lour.) pers. *Journal of Food Protection*.

[62] Chakrabarti R., Singh B. A., Vn P., Vanchhawng L. T., Thirumurugan K. (2014). Screening of nine herbal plants for in vitro *α*-amylase inhibition. *Asian Journal of Pharmaceutical and Clinical Research*.

[63] Jaykumar A. B., Shihabudeen M. S., Thirumurugan K. (2011). Screening of fifteen Indian ayurvedic plants for alpha-glucosidase inhibitory activity and enzyme kinetics. *International Journal of Pharmacy and Pharmaceutical Sciences*.

[64] Vyas B., Rana A. C. (2013). Phytopharmacological evaluation of hydro-alcoholic extract of *Mesua ferrea* stamen for their anti-oxidant and antidiabetic activity. *Journal of Chemical and Pharmaceutical Research*.

[65] Balekari U., Veeresham C., Veeresham C. (2015). Insulinotropic activity of methanolic extract of *Mesua ferrea* Linn. *Journal of Basic & Applied Sciences*.

[66] Asif M., Jafari S. F., Iqbal Z. (2017). Ethnobotanical and Phytopharmacological attributes of *Mesua ferrea*: a mini review. *Journal of Applied Pharmaceutical Science*.

[67] Rouger C., Derbré S., Richomme P. (2018). *Mesua* sp.: chemical aspects and pharmacological relevance of prenylated polyphenols. *Phytochemistry Reviews*.

