# Supplementary material for: Evaluation on Antidiabetic Properties of Medicinal Plants from Myanmar
Source: ScientificWorldJournal. 2021 Aug 26;2021:1424675. doi: 10.1155/2021/1424675 (PMC8423552; doi:10.1155/2021/1424675)
Supplement: Supplementary Materials — Table S1. Information with 30–50% PPL inhibition activities of 17 medicinal plants. Table S2. 82 species of 51 families with antidiabetic properties research status. Table S3. Relative plants in antidiabetic reports without any antidiabetes research. Table S4. The information of antidiabetic ingredients. Figure S1. Antidiabetic components from medicinal plants in Myanmar. [file 1424675.f1.docx]

Evaluation on antidiabetic properties of medicinal plants from Myanmar

Dongdong Zhang^1,2,3^, Karuppusamy Arunachalam^1^, Yuehu Wang ^1^, Yu Zhang^1^, Jun Yang^1^, Pyae Phyo Hein^1,3^, Aye Mya Mon^1,3^, Jianwen Li^1^, Angkhana Inta^2*^, and Xuefei Yang^1,3^*

^1^Key Laboratory of Economic Plants and Biotechnology and the Yunnan Key Laboratory for Wild Plant Resources, Kunming Institute of Botany, Chinese Academy of Sciences, Kunming, 650201, People’s Republic of China

^2^Department of Biology, Faculty of Science, Chiang Mai University, 239 Huay Kaew Road, Chiang Mai 50200, Thailand

^3^Southeast Asia Biodiversity Research Institute, Chinese Academy of Sciences, Yezin, Nay Pyi Taw 05282, Myanmar

Table S1 Information with 30-50% PPL inhibition activities of 17 medicinal plants

| **No.** | **Scientific name** | **Family** | **Myanmar name** | **Part**^a^ | **Traditional use in Myanmar** | PPL inhibition (%) |
| --- | --- | --- | --- | --- | --- | --- |
| MY17 | *Crateva religiosa* G.Forst. | Capparaceae | Lè-seik-shin | Barks | A paste from grinding the bark together with paranawar (*Boerhavia diffusa*) root is taken to cure chronic sores and boils [1] | 46.91 ± 2.37 |
| MY70 | *Cyperus scariosus* R.Br. | Cyperaceae | Nwar myay yinn | Roots | Phlegm rancidness phlegm, bile, fever and bowel problems, appetite, thirst, burning sensation, asthma, venomous snakebites, nausea, gastric ailments, sour stomach, swollen limbs, itching, leprosy, herpes, and scabies [1] | 46.85 ± 0.37 |
| MY68 | *Pterocarpus santalinus* L.f. | Fabaceae | Natha-ni | Woods | Fever, bowel disorder, giddiness, heart palpitation, insomnia, jaundice, cold and cough, smallpox and measles, heart prostration, blood and bile disorder,malaria [2] | 46.05 ± 0.06 |
| MY27 | *Kaempferia galanga* L. | Zingiberaceae | Kun - sa - gamon | Rhizomes | Poor appetite in digestion for general well-being [3] | 45.98 ± 2.26 |
| MY10 | *Mesua ferrea* L. | Calophyllaceae | Guntgaw | Flowers | Astringent,coughs, stomach problems, and excessive perspiration and phlegm, ground together with thanakha (*Hesperethusa crenulata)* for boils and other skin conditions [1] | 45.01 ± 0.94 |
| MY11 | *Myrica nagi* Thunb. | Myricaceae | Kat-pho | Barks | Curing running nose, cough, toothache, earache, asthma, diarrhoea, liver complaints, piles, epilepsy and wounds [4] | 42.49 ± 1.96 |
| MY89 | *Centella asiatica* (L.) Urb. | Apiaceae | Myin-hkwa | Whole plants | Treat diabetes, and as a laxative and diuretic [1] | 39.92 ± 0.52 |
| MY1 | *Antidesma acidum* Retz. | Phyllanthaceae | Chin-pyone, Ma-gan-pyan | Aerial parts | Urinary disorder (Shan State in Myamar) | 38.28 ± 1.21 |
| MY9 | *Eriobotrya bengalensis* Kurz | Rosaceae | Maksawt, Pet-sut | Leaves | Undocumented | 37.70 ± 1.53 |
| MY66 | *Soymida febrifuga* (Roxb.) A. Juss. | Meliaceae | Dant-tagu-ni | Woods | Undocumented | 37.18 ± 2.42 |
| MY18 | *Cajanus volubilis* (King) Maesen | Fabaceae | Mon-hto-sone (Shan name) | Aerial parts | The whole plant is boiled in the hot water and taken shower for inflammation and hives (Shan State in Myamar) | 34.56 ± 0.94 |
| MY67 | *Curcuma comosa* Roxb. | Zingiberaceae | Nanwinga | Roots | Mixed with honey is taken twice daily to lower blood pressure[1] | 33.22 ± 0.84 |
| MY31 | *Mimusops elengi* L. | Sapotaceae | Thitcho-khaya | Flowers | Cough, treating white vaginal discharge and dental diseases[1] | 31.32 ± 1.01 |
| MY26 | *Canscora andrographioides* Griff. ex C.B.Clarke | Gentianaceae | Hin-khar | Whole plants | Stomachache | 30.94 ± 1.21 |
|  | Positive control: Orlistat (0.005 μg/mL) | | | | | 88.85 ± 0.82 |

Table S2 Research status of selected plants in anti-diabetes

| **NO.** | **Species** | **Family** | **Parts used** | **Effects reported** | | **Ref.** |
| --- | --- | --- | --- | --- | --- | --- |
| MY55 | *Andrographis paniculata* (Burm.f.) Nees | Acanthaceae | Whole plants | dry powder of aerial part significantly lowered HbA1c and fasting serum insulin in patients with type 2 diabetes without induced significant adverse | [5] | |
| MY96 | *Acorus calamus* L. | Acoraceae | Rhizomes | Antihyperglycemic (rats) | [6] | |
| MY22 | *Allium sativum* L. | Amaryllidaceae | Bulbs | Significant hypoglycemic and hypolipidemic effects in type 2 diabetic patients | [7] | |
| MY30 | *Rhus chinensis* Mill. | Anacardiaceae | Fruits | Pancreatic lipase inhibitory (IC_50_ 59-63 μg/ml) | [8] | |
| MY89 | *Centella asiatica* (L.) Urb. | Apiaceae | Whole plants | Antidiabetic (rats) | [9] | |
| MY44 | *Foeniculum vulgare* Mill. | Apiaceae | Seeds | Antidiabetic (rats) | [10] | |
| MY32 | *Carissa spinarum* L. | Apocynaceae | Aerial parts | Treatment of diabetes in Benin | [11] | |
| MY95 | *Holarrhena pubescens* Wall. ex G.Don | Apocynaceae | Stem barks | Antioxidant and antidiabetic (mice) | [12] | |
| MY62 | *Plumeria rubra* L. | Apocynaceae | Flowers | Antidiabetic and hypoglycaemic (rats) | [13] | |
| MY84 | *Rauvolfia serpentina* (L.) Benth. ex Kurz(L.) | Apocynaceae | Roots | Hypoglycemic and hypolipidemic (rats) | [14] | |
| MY108 | *Tylophora indica* (Burm. f.) Merr. | Apocynaceae | Stems | Antidiabetic (leaves, rats) | [15] | |
| MY99 | *Amorphophallus paeoniifolius* (Dennst.) Nicolson | Araceae | Rhizomes | To prevent the formation of excess fat and solidify fatty deposits in the body in Myanmar | [1] | |
| MY34 | *Metapanax delavayi* (Franch.) J.Wen & Frodin | Araliaceae | Leaves | Aldose reductase inhibition (IC_50_ 17.53 μg/ml) | [16] | |
| MY107 | *Aristolochia indica* L. | Aristolochiaceae | whole plants | Hypoglycemic (mice) | [17] | |
| MY25 | *Anacyclus pyrethrum* (L.) Lag. | Asteraceae | roots | Antidiabetic, α‑amylase inhibition (IC_50_ < 60 μg/ml) | [18,19] | |
| MY24 | *Oroxylum indicum* (L.) Kurz | Bignoniaceae | Stem | α-Glucosidase (IC_50_ 31.9 μg/ml) | [20] | |
| MY46 | *Lepidium sativum* L. | Brassicaceae | Seeds | Hypoglycemic (rats) | [21] | |
| MY10 | *Mesua ferrea* L. | Calophyllaceae | Flowers | Antidiabetic (rats), α-glucosidase (IC_50_ 129 μg/ml), α‑amylase inhibitory (IC_50_ 146.8 μg/ml) | [22-24] | |
| MY17 | *Crateva religiosa* G.Forst. | Capparaceae | Stems | Roots are taken to treat diabetes and kidney stones after boiled in water in Myanmar | [1] | |
| MY15 | *Valeriana jatamansi* Jones | Caprifoliaceae | Rhizomes | Restore blood lipids level，and protect liver (rats) | [25] | |
| MY86 | *Garcinia pedunculata* Roxb. ex Buch.-Ham. | Clusiaceae | Fruits | Antihyperglycemic, antidiabetic, and antioxidant (rats) | [26] | |
| MY23 | *Eclipta prostrata* (L.) L. | Compositae | Whole plants | Antihypoglycemic (rats), α-glucosidase inhibition in a noncompetitive manner and eye lens aldose reductase with IC_50_ values of around 54 and 4.5 μg/ml, respectively | [27] | |
| MY37 | *Chromolaena odorata* (L.) R.M.King & H.Rob. | Compositae | aerial parts | Antidiabetic (leaves, mice) | [28] | |
| MY78 | *Saussurea costus* (Falc.) Lipsch. | Compositae | roots | Lipase and amylase inhibition | [29] | |
| MY39 | *Tithonia diversifolia* A. Gray. | Compositae | flowers | Leaves showed antidiabetic effects via modulating GLUT2 expression (100, 200, and 300 mg/kg b.w., mice) | [30] | |
| MY43 | *Cuscuta reflexa* Roxb. | Convolvulaceae | whole plants | Antidiabetic (rats) | [31] | |
| MY70 | *Cyperus scariosus* R.Br. | Cyperaceae | Roots | Lipid lowering and antioxidant (pigs) | [32] | |
| MY2 | *Croton tiglium* L. | Euphorbiaceae | Seeds | α-Amylase inhibition (IC_50_ 39.4 μg/ml) | [33] | |
| MY72 | *Baliospermum solanifolium* (Burm.) Suresh | Euphorbiaceae | Stems | roots showed antidiabetic effects (rats, 200 mg/kg) | [34] | |
| MY93 | *Abrus precatorius* L. | Fabaceae | Seeds | Hypoglycemic and hypolipidemic (rats) | [35] | |
| MY47 | *Acacia farnesiana* (L.) Willd. | Fabaceae | flowers | Leaves for anti-diabetes in India | [36] | |
| MY6 | *Caesalpinia digyna* Rottler | Fabaceae | Seeds | Antidiabetic activity (roots, rats) | [37] | |
| MY71 | *Glycyrrhiza glabra* L. | Fabaceae | Roots | Antihyperglycemic and antihyperlipidemic (rats) | [38] | |
| MY103 | *Pongamia pinnata* Pierre | Fabaceae | stems | Antihyperglycemic (stem bark, mice) | [39] | |
| MY68 | *Pterocarpus santalinus* L.f. | Fabaceae | Woods | Glucose uptake (C2C12 cell line) | [40] | |
| MY77 | *Trigonella foenum-graecum* L. | Fabaceae | Seeds | safe and efficacious in ameliorating the symptoms of T2D in humans | [41,42] | |
| MY33 | *Entada phaseoloides* (L.) Merr. | Fabaceae | Seeds | Hypoglycemic and hypolipidemic (total saponins, rats) | [43] | |
| MY51 | *Mimosa pudica* L. | Fabaceae | Aerial parts | Treatment of diabetes in India  Antidiabetic activity in induced diabetic mice (200 mg/kg b.w.) | [44] | |
| MY81 | *Senna siamea* (Lam.) H.S.Irwin & Barneby | Fabaceae | Leaves | α-Glucosidase inhibition (83%, 25 μg/mL), α-amylase inhibition (82%, 25 μg/mL) | [45] | |
| MY91 | *Swertia angustifolia* Buch.-Ham. ex D. Don | Gentianaceae | Whole Plants | Infusion for diabetes in India. Decoction for diabetes in Myanmar (Shan State by survey) | [46] | |
| MY54 | *Clerodendrum bungei* Steud. | Lamiaceae | unknown | α-Glucosidase inhibition (IC_50_ 114.89 μg/ml) | [47] | |
| MY49 | *Thymus vulgaris* L. | Lamiaceae | Seeds | Anti-hyperglycemic and anti-hyperlipidemic (leaves, rats) | [48] | |
| MY63 | *Vitex trifolia* L. | Lamiaceae | aerial parts | Up-regulates lipid accumulation and adipogenesis in 3T3-l1 preadipocytes (leaves) | [49] | |
| MY104 | *Cinnamomum bejolghota* (Buch.-Ham.) Sweet | Lauraceae | Barks | Antihyperglycemic (rats) | [50] | |
| MY5 | *Cinnamomum tamala* (Buch.-Ham.) T.Nees & Eberm. | Lauraceae | leaves | Antidiabetic and anti-dyslipidemic (rats) | [51,52] | |
| MY4 | *Litsea cubeba* (Lour.) Pers. | Lauraceae | Fruits | α-Amylase inhibition (IC_50_ 514.9 µg/ml) | [53] | |
| MY29 | *Strychnos potatorum* L. f. | Loganiaceae | Seeds | Antidiabetic (rats) | [54] | |
| MY98 | *Lagerstroemia speciosa* (L.) Pers. | Lythraceae | Aerial parts | Leaves extracts contain 1% corosolic acid can balance blood sugar in humans | [55] | |
| MY97 | *Grewia tiliifolia* Vahl | Malvaceae | Barks | Antidiabetic, antihyperlipidemic (rats) | [56] | |
| MY36 | *Martynia annua* L. | Martyniaceae | Fruits | α-Glucosidase inhibition (IC_50_ 78.8 µg/ml) | [57] | |
| MY106 | *Azadirachta Indica* A. Juss. | Meliaceae | Leaves | Seed possess hypocholesterolemic properties in T2DM patients | [58] | |
| MY66 | *Soymida febrifuga* (Roxb.) A. Juss. | Meliaceae | Woods | Methanolic bark extract possess hypoglycemic and anti-hyperlipidemic (200 mg/kg, rats) | [59] | |
| MY64 | *Cissampelos pareira* L. | Menispermaceae | Aerial part | Hypoglycemic activity and slight toxicity (leaves, rats) | [60] | |
| MY105 | *Coscinium fenestratum* (Goetgh.) Colebr. | Menispermaceae | Stems | Antidiabetic (rats) | [61] | |
| MY57 | *Tinospora sinensis* (Lour.) Merr. | Menispermaceae | Stems | Antihyperglycemic (rats), α-amylase and α-glucosidase | [62,63] | |
| MY12 | *Glinus oppositifolius* (L.) A. DC. | Molluginaceae | Whole plants | Antihyperglycemic (rats) | [64] | |
| MY11 | *Myrica nagi* Thunb. | Myricaceae | Barks | Barks showed α-amylase (IC_50_ 47.5 µg/ml), fruits showed α-amylase (IC_50_ 60 µg/ml), α-glucosidase (IC_50_ 100 µg/ml), inhibitory activity against lipase (IC_50_ 91 µg/ml) | [65,66] | |
| MY61 | *Myristica fragrans* Houtt. | Myristicaceae | Seeds | Antidiabetic (rats) | [67] | |
| MY94 | *Syzygium aromaticum* (L.) Merr. & L.M. Perry | Myrtaceae | Flowers | Glucose lowering effect (rats), α-glucosidase and α-amylase inhibition (IC_50_ 0.3 and 36.2 µg/ml, respectively) | [68,69] | |
| MY73 | *Nelumbo nucifera* Gaertn. | Nelumbonaceae | Flowers | Lowering blood glucose level (rats) | [70] | |
| MY1 | *Antidesma acidum* Retz. | Phyllanthaceae | Aerial parts | Boiled extract of leaves for diabetes in India | [71] | |
| MY42 | *Piper nigrum* L. | Piperaceae | Fruits | Anti-hypolipidemic (rats) | [72] | |
| MY80 | *Piper retrofractum* Vahl | Piperaceae | Fruits | Diabetes treatment in Thai | [73] | |
| MY60 | *Picrorhiza kurroa* Royle | Plantaginaceae | Rhizomes | Displayed β-cell regeneration with enhanced insulin production and antihyperglycemic effects (rats) | [74] | |
| MY14 | *Plumbago zeylanica* L. | Plumbaginaceae | Stems | Root extract showed hypoglycemic, hepatoprotective (100, 200 mg/kg, rats) | [75] | |
| MY111 | *Cynodon dactylon* (L.) Pers. | Poaceae | Aerial parts | Antihyperglycemic (leaves, rats) | [76] | |
| MY53 | *Aconitum ferox* Wall. ex Ser. | Ranunculaceae | Roots | Antihyperglycemic (rats) | [77] | |
| MY109 | *Coptis teeta* Wall. | Ranunculaceae | rhizomes | Water extract for diabetes treatment in India | [78] | |
| MY48 | *Nigella sativa* L. | Ranunculaceae | Seeds | Significant activity in diabetic and dyslipidemic patients | [79,80] | |
| MY85 | *Carallia brachiata* (Lour.) Merr. | Rhizophoraceae | Barks | Hypoglycemic activity (leaves, rats) | [81] | |
| MY50 | *Oldenlandia corymbosa* L. | Rubiaceae | whole plants | Decoction lower blood sugar in Thailand | [82] | |
| MY112 | *Harrisonia perforata* (Blanco) Merr. | Rutaceae | Leaves | Decoction roots lower blood sugar in Thailand | [82] | |
| MY69 | *Santalum album* L. | Santalaceae | Woods | Antihyperlipidemic activity (rats) | [83] | |
| MY8 | *Sapindus rarak* DC. | Sapindaceae | pericarps | Pancreatic lipase inhibitory activity (IC_50_ = ca. 614 μg/mL) | [84] | |
| MY31 | *Mimusops elengi* L. | Sapotaceae | flowers | Antihyperglycemic (barks, 200, 400 mg/kg, mice) | [85] | |
| MY90 | *Brucea javanica* (L.) Merr. | Simaroubaceae | Seeds | Antihyperglycemic and antioxidant (rats) | [86] | |
| MY113 | *Leea asiatica* (L.) Ridsdale | Vitaceae | Leaves | Treat diabetes traditionally in India | [87] | |
| MY82 | *Amomum subulatum* Roxb. | Zingiberaceae | Fruits | Traditionally for diabetes treatment | [88] | |
| MY59 | *Boesenbergia rotunda* (L.) Mansf. | Zingiberaceae | Roots | α-Glucosidase and pancreastic lipase | [89] | |
| MY67 | *Curcuma comosa* Roxb. | Zingiberaceae | rhizomes | Improved glucose and lipid metabolism (rats) | [90] | |
| MY27 | *Kaempferia galanga* L. | Zingiberaceae | rhizomes | Antidiabetic (rats) | [91] | |
| MY38 | *Zingiber officinale* Roscoe | Zingiberaceae | rhizomes | Improved insulin sensitivity (DM2 patients) | [92] | |

Figure S1 Antidiabetic components from medicinal plants in Myanmar

Figure S1 Antidiabetic components from medicinal plants in Myanmar


Figure S1 Antidiabetic components from medicinal plants in Myanmar

Figure S1 Antidiabetic components from medicinal plants in Myanmar

Table S3 The relative plants of selected species on anti-diabetic reports as it without any anti-diabetes research

| **NO.** | **Family** | **Species screened** | **Parts used** | **Reported species effects** | **Ref.** |
| --- | --- | --- | --- | --- | --- |
| MY16 | Apiaceae | *Ligusticum officinale* (Makino) Kitag. | Rhizomes | Roots of *L. porter* showed significant hypoglycemic and antihyperglycemic (mice) | [93] |
| MY35 | Araceae | *Caladium lindenii* (André) Madison | Rhizomes | Corm of *C. bicolor* showed antidiabetic and hepatoprotective activity on Hep G2 cells | [94] |
| MY40 | Aristolochiaceae | *Aristolochia tagala* Cham. | Roots | *A. indica* roots showed antihyperglycemic effect (200, 400 mg/kg, rats) | [95] |
| MY114 | Berberidaceae | *Mahonia* sp. | Aerial parts | *M. nepalensis* have potent antidiabetic activity in streptozocin induced diabetic rats (200 and 400 mg/kg) | [96] |
| MY58 | Boraginaceae | *Heliotropium ovalifolium* Forssk. | Aerial parts | *H. indicum* leaves showed anti-hyperglycaemic and anti-dyslipidemic activities (30 and 75 mg/kg b. wt., rats) | [97] |
| MY102 | Capparaceae | *Boscia variabilis* Collett & Hemsl. | Stems | *B. senegalensis* seeds possess anti-hyperglycaemic effect (250 mg/kg, albino rabbits) | [98] |
| MY21 | Combretaceae | *Terminalia citrina* (Gaertn.) Roxb. | Fruits | *T. chebula* fruits showed α-glucosidase inhibitory activity (IC_50_ = 38.2 μg/ml), *T. catappa* fruits showed antidiabetic (rats) | [99,100] |
| MY87 | Convolvulaceae | *Argyreia barbigera* Choisy | Roots | Silver nanosynthesis with *A. nervosa* leaves inhibited α-amylase and α-glucosidase activities (EC_50_ = 55.5 and 51.7 μg/mL, respectively) | [101] |
| MY18 | Fabaceae | *Cajanus volubilis* (King) Maesen | Aerial parts | Seed flour of *C. cajan* reduced blood glucose level (rats) | [102,103] |
| MY76 | Fabaceae | *Pterocarpus indicus* Willd. | Woods | Heartwood of *P. marsupium* can lower the blood lower of T2DM paients | [104] |
| MY20 | Gentianaceae | *Canscora diffusa* (Vahl) R.Br. ex Roem. & Schult. | Leaves | Hypoglycaemic effect of whole plants of *C. decussate* (400 and 600 mg/kg, rabbits) | [105,106] |
| MY26 | Gentianaceae | *Canscora andrographioides* Griff. ex C.B.Clarke | Aerial parts | *C. perfoliata* whole plant showed hypoglycemic and hypolipidemic effects (150 and 300 mg/kg, rats) | [103] |
| MY110 | Rubiaceae | *Mitragyna parvifolia* (Roxb.) Korth. | Flowers | *M. speciose* leaves stimulates glucose uptake (L8 muscle cell) | [107] |

Table S4 Anti-diabetic ingredients of selected medicinal plants from Myanmar

| **No** | **Compound names** | **Types** | **Sources of plants** | **Anti-diabetic activities** | **Ref.** |
| --- | --- | --- | --- | --- | --- |
| **1** | Tagitinin G | Sesquiterpene | *Tithonia diversifolia* A. Gray. | Increase glucose uptake (35.46 μM) | [108] |
| **2** | Tagitinin I | Sesquiterpene |  | Increase glucose uptake (29.94 μM) | [108] |
| **3** | 1*β*-Hydroxydiversifolin- 3-*O*-methyl ether | Sesquiterpene |  | Increase glucose uptake (27.32 μM) | [108] |
| **4** | Tirotundin | Sesquiterpene |  | PPARγ transactivation activity (IC_50_ 27 μM) | [109] |
| **5** | Andrographolide | Diterpenoid | *Andrographis paniculata* (Burm.f.) Nees | Diabetic rat model (1.5 and 2 mg/kg) | [110] |
| **6** | Nimbidiol | Diterpenoid | *Azadirachta Indica* A. Juss. | Intestinal maltase (IC_50_ 12 μM), intestinal sucrose (IC_50_ 6.75 μM)  Intestinal lactase (IC_50_ 20 μM)  Intestinal trehalase ((IC_50_ 30 μM)) | [111] |
| **7** | Meliacinolin | Triterpenoid |  | Antidiabetic activity in STZ-diabetic mice (20 mg/kg b.w.) | [112] |
| **8** | Lupenone | Triterpenoid | *Abrus precatorius* L. | α-amylase inhibition (IC_50_ 31 μM) | [113] |
| **9** | Monogynol A | Triterpenoid | *Plumeria rubra* L. | PTP1B inhibition (IC_50_ 9.34 μM) | [114] |
| **10** | (20*R*)-3-oxolupan-30-al | Triterpenoid |  | PTP1B inhibition (IC_50_ 6.39 μM) | [114] |
| **11** | Lup-20(29)-en-3*β*-[1-(2*S*-hydroxypropionate)-benzoic acid] | Triterpenoid |  | α-glucosidase inhibition (IC_50_ 19.45 μM)  PTP1B inhibition (IC_50_ 0.21 μM) | [114] |
| **12** | Cycloart-23-ene-3*β*,25-diol | Triterpenoid | *Pongamia pinnata* Pierre | Antidiabetic activity in STZ–nicotinamide induced diabetic mice (10 mg/kg, p.o.) | [115] |
| **13** | Entagenic acid | Triterpenoid | *Entada phaseoloides* (L.) Merr. | Antidiabetic effect in T2DM rats (5, 10, 20 mg/kg) | [116] |
| **14** | Eclalbasaponin II | Triterpenoid | *Eclipta prostrata* (L.) L. | PTP1B Inhibition (IC_50_ 11.75 μM) | [117] |
| **16** | Eclalbasaponin III | Triterpenoid |  | PTP1B Inhibition (IC_50_ 15.23 μM) | [117] |
| **16** | Eclalbasaponin V | Triterpenoid |  | PTP1B Inhibition (IC_50_ 10.88 μM) | [117] |
| **17** | Oleanolic acid | Triterpenoid | *Lagerstroemia speciosa* (L.) Pers. | α-glucosidase inhibition (IC_50_ 13.76µM) | [118] |
| **18** | Arjunolic acid | Triterpenoid |  | α-glucosidase inhibition (IC_50_ 38.10 µM) | [118] |
| **19** | Maslinic acid | Triterpenoid |  | α-glucosidase inhibition (IC_50_ 11.67 µM) | [118] |
| **20** | 22*β*-Acetoxyolean-12-ene-3*β*, 24-diol | Triterpenoid | *Trigonella foenum-graecum* L. | α-glucosidase inhibition (IC_50_ 15.16 µM) | [119] |
| **21** | Soyasapogenol B | Triterpenoid |  | α-glucosidase inhibition (IC_50_ 8.98 µM) | [119] |
| **22** | Corosolic acid | Triterpenoid | *Lagerstroemia speciosa* (L.) Pers. | α-glucosidase inhibition (IC_50_ 7.46 µM) | [118] |
| **23** | 23-Hydroxyursolic acid | Triterpenoid |  | α-glucosidase inhibition (IC_50_ 17.21 µM) | [118] |
| **24** | Asiatic acid | Triterpenoid | *Centella asiatica* (L.) Urb. | Antidiabetic activities in diabetic rats (40 mg/kg b.w.) And goto-kakizaki rats (40 mg/kg) | [120,121] |
| **25** | Brucein A | Triterpenoid | *Brucea javanica* (L.) Merr. | Lipolytic activity in 3T3-L1 adipocytes (10 µM) | [122] |
| **26** | Brusatol | Triterpenoid |  | Lipolytic activity in 3T3-L1 adipocytes (10 µM) | [122] |
| **27** | Bruceantinol | Triterpenoid |  | Lipolytic activity in 3T3-L1 adipocytes (10 µM) | [122] |
| **28** | Brucein B | Triterpenoid |  | Lipolytic activity in 3T3-L1 adipocytes (10 µM) | [122] |
| **29** | 3′-Hydroxybrucein A | Triterpenoid |  | Lipolytic activity in 3T3-L1 adipocytes (10 µM) | [122] |
| **30** | Brucein C | Triterpenoid |  | Lipolytic activity in 3T3-L1 adipocytes (10 µM) | [122] |
| **31** | Bruceine E | Triterpenoid |  | Blood glucose lowering effect in both the normoglycemic  Mice and STZ induced diabetic rats (2 mg/kg) | [123] |
| **32** | bruceine D | Triterpenoid |  | Blood glucose lowering effect in both the normoglycemic  Mice and STZ induced diabetic rats (2 mg/kg) | [123] |
| **33** | Berberine | Alkaloid | *Coptis teeta* Wall. | Effect on treatment type 2 DM patients in clinical trials | [124,125] |
| **34** | Coptisine | Alkaloid | *Coptis chinensis* Franch. | Inhibition of cellular triglyceride accumulation in 3T3-L1 adipocytes (IC_50_ 39.2 μM) | [126] |
| **35** | Piperine | Alkaloid | *Piper nigrum* L. | Anti-hyperglycemic activity alloxan-induced diabetic rats (25 mg/kg) | [127] |
| **36** | Neferine | Alkaloid | *Nelumbo nucifera* Gaertn. | Decreased fasting glucose and insulin resistance, ameliorated memory and cognitive dysfunction in db/db diabetic mice (25, 50 mg/ml) possibly through modulating NLRP3 inflammasome pathway and alleviating ER stress | [128] |
| **37** | Isoliensinine | Alkaloid |  | Improving the Symptoms of T2DM mice via Activation of AMP-Activated Kinase and Regulation of PPARγ (30 mg/kg) | [129] |
| **38** | 17-*O*-(*β*-D-glucopyranosyl)-4-*O*-methylnigellidine | Alkaloid | *Nigella sativa* L. | Regulate glucose consumption by activation of AMPK (25 μM) | [130] |
| **39** | Oroxylin A | Flavonoid | *Oroxylum indicum* (L.) Kurz | α-glucosidase inhibition (IC_50_ 27.91 μM) | [20] |
| **40** | Oroxyloside | Flavonoid |  | α-glucosidase inhibition (IC_50_ 2.13 μM) | [20] |
| **41** | Hispidulin | Flavonoid |  | α-glucosidase inhibition (IC_50_ 7.03 μM) | [20] |
| **42** | Apigenin | Flavonoid | *Eclipta prostrata* (L.) L. | PTP1B Inhibition (IC_50_ 45.50 μM) | [117] |
| **43** | Hesperetin-7-*O*-*β*-D-glucoside | Flavonoid |  | PTP1B Inhibition (IC_50_ 14.11 μM) | [117] |
| **44** | Kaempferol 3-*O*-rutinoside | Flavonoid | *Nigella sativa* L. | α-glucosidase inhibition (IC_50_ 32.7 μM) | [131] |
| **45** | licoflavanone A | Flavonoid | *Glycyrrhiza glabra* L. | PPAR-γ-ligand-binding activity (31.06 µM) | [132] |
| **46** | (2*R*,3*R*)-3,4′,7-trihydroxy-3′-prenylflavane | Flavonoid |  | PPAR-γ-ligand-binding activity (29.41 µM) | [132] |
| **47** | Glabrol | Flavonoid |  | PPAR-γ-ligand-binding activity (25.51 µM) | [132] |
| **48** | Shinflavanone | Flavonoid |  | PPAR-γ-ligand-binding activity (25.64 µM) | [132] |
| **49** | Gancaonin L | Flavonoid |  | PPAR-γ-ligand-binding activity (28.24 µM) | [132] |
| **50** | Glabrone | Flavonoid |  | PPAR-γ-ligand-binding activity (29.76 µM) | [132] |
| **51** | 5′-formylglabridin | Isoflavans |  | PPAR-γ-ligand-binding activity (28.41 µM) | [132] |
| **52** | (3*R*)-2′,3′,7-trihydroxy-4′-methoxyisoflavan | Isoflavans |  | PPAR-γ-ligand- binding activity (34.72 µM) | [132] |
| **53** | Kanzonol X | Isoflavans |  | PPAR-γ-ligand-binding activity (29.76 µM) | [132] |
| **54** | Shinpterocarpin | Pterocarpans |  | PPAR-γ-ligand-binding activity (31.06 µM) | [132] |
| **55** | *trans*-resveratrol | Chalcones | *Senna siamea* (Lam.) H.S.Irwin & Barneby | α-Glucosidase inhibition (IC_50_ 6.57 μM) | [133] |
| **56** | Piceatannol | Chalcones |  | α-Glucosidase inhibition (IC_50_ 4.76 μM) | [133] |
| **57** | Dihydropiceatannol | Chalcones |  | α-Glucosidase inhibition (IC_50_ 13.09 μM) | [133] |
| **58** | Echinatin | Chalcones | *Glycyrrhiza glabra* L. | PPAR-γ-ligand-binding activity (37.04 µM) | [132] |
| **59** | 2',4' ,6'-trihydroxydihydrochalcone | Chalcones | *Boesenbergia rotunda* (L.) Mansf. | α-Glucosidase inhibition (IC_50_ 32.0 μM) | [89] |
| **60** | Panduratin A | Chalcones |  | α-Glucosidase inhibition (IC_50_ 12.7 μM), lipase inhibitory (IC_50_ 17.1 μM) | [89] |
| **61** | Isopanduratin A | Chalcones |  | α-Glucosidase inhibition (IC_50_ 7.5 μM), lipase inhibitory (IC_50_ 15.1 μM) | [89] |
| **62** | Hydroxypanduratin | Chalcones |  | α-Glucosidase inhibition (IC_50_ 4.6 μM) | [89] |
| **63** | Rotundaflavanochalcone | Chalcones |  | α-Glucosidase inhibition (IC_50_ 2.4 μM), lipase inhibitory (IC_50_ 25.8 μM) | [89] |
| **64** | *iso*-rotundaflavanochalcone | Chalcones |  | α-Glucosidase inhibition (IC_50_ 3.4 μM), lipase inhibitory (IC_50_ 30.1 μM) | [89] |
| **65** | de-*O*-methyl rotundaflavanochalcone | Chalcones |  | α-Glucosidase inhibition (IC_50_ 1.3 μM), lipase inhibitory (IC_50_ 10.6 μM) | [89] |
| **66** | Cinnamtannin D1 | Biflavonoid | *Cinnamomum tamala* (Buch.-Ham.) T.Nees & Eberm. | Hypoglycemic activity in diabetic db/db mice (20 mg/kg) by AMPK/mtor/ULK1 pathway activate autophagy | [134] |
|  |  |  |  |  |  |
| **67** | *meso*-dihydroguaiaretic acid | Lignans | *Myristica fragrans* Houtt. | PTP1B inhibition (IC_50_ 19.6 μM) | [135] |
| **68** | Otobaphenol | Lignans |  | PTP1B inhibition (IC_50_ 48.9 μM) | [135] |
| **69** | Tetrahydrofuroguaiacin B | Lignans |  | Stimulation AMPK in differentiated C2C12 cells (5 μM) | [136] |
| **70** | Nectandrin B | Lignans |  | Stimulation AMPK in differentiated C2C12 cells (5 μM) | [136] |
| **71** | Nectandrin A | Lignans |  | Stimulation AMPK in differentiated C2C12 cells (5 μM) | [136] |
| **72** | Macelignan | Lignans |  | Enhanced insulin sensitivity and improved lipid metabolic disorders by activating PPARα/γ and attenuating ER stress (25 mg/kg) | [137] |
| **73** | Cysestermerol A | Stilbene | *Cynodon dactylon* (L.) Pers. | Increased the glucose consumption in hepg2 cells (12.5 µM) | [138] |
| **74** | Kanzonol W | 3-arylcoumarin | *Glycyrrhiza glabra* L. | PPAR-γ-ligand-binding activity (29.76 µM) | [132] |
| **75** | Bergenin | Coumarin | *Caesalpinia digyna* Rottler | T2DM diabetic rats (10 mg/kg p.o.) | [139] |
| **76** | Emodin | Quinones | *Senna siamea* (Lam.) H.S.Irwin & Barneby | PTP1B inhibition (IC_50_ 12.48 μM) | [133] |
| **77** | Thymoquinone | Quinones | *Nigella sativa* L. | Increased endogenous GLP-1 levels to reduce hyperphagy in STZ -diabetic rats (25 mg/kg) | [140,141] |
| **78** | Plumbagin | Naphthoquinones | *Plumbago zeylanica* L. | STZ-induced diabetic rats (15 and 30 mg/kg b. W.) | [142] |
| **79** | Protocatechuic acid | Phenylpropanoids | *Oroxylum indicum* (L.) Kurz | α-glucosidase inhibition (IC_50_ 45.32 μM) | [20] |
| **80** | 4-hydroxybenzoic acid | Phenylpropanoids | *Eclipta prostrata* (L.) L. | PTP1B Inhibition (IC_50_ 48.70 μM) | [117] |
| **81** | Apocynin | Phenylpropanoids | *Picrorhiza kurroa* Royle | Anti-diabetic and anti-adipogenic effects by regulating resist insulin in vitro (1 μM), ameliorated biochemical indexes and diabetic retinopathy and inhibited of TLR4/NF-κB signaling pathway activity in STZ diabetic rats (16 mg/kg) | [143,144] |
| **82** | Myoinositol | Alcohols | *Mimosa pudica* L. | Antidiabetic activity in HFD fed-STZ-induced insulin-resistant diabetic rats (25 and 50 mg/kg) | [145] |
| **83** | (2*S*, 3*R*, 4*S*) 4-hydroxyisoleucine | Amino acid | *Trigonella foenum-graecum* L. | Anti-diabetic activities in type I diabetes, STZ-treated rats (50 mg/kg) | [146] |
| **84** | (2*S*)-1-*O*-stearoyl-3-*O*-*β*-D-galactopyranosyl-*sn*- glycerol | Fatty acid | *Eclipta prostrata* (L.) L. | PTP1B Inhibition (IC_50_ 2.14 μM) | [117] |
| **85** | (2*S*)-3-*O*-(9*Z*,12*Z* octadecadienoyl) -glyceryl-*O*-*β*-D-galactopyranoside | Fatty acid |  | PTP1B Inhibition (IC_50_ 3.21 μM) | [117] |
| **86** | Isonarthogenin | Steroid | *Trigonella foenum-graecum* L. | α-glucosidase inhibition (IC_50_ 7.26 µM) | [119] |
| **87** | (25*R*)-5-en-spirostane-3*β*-ol 3-*O*-*β*-D-glucopyranosyl-(1→4)-*β*-D-glucopyranoside | Steroid |  | α-glucosidase inhibition (IC_50_ 5.49 µM) | [119] |
| **88** | (25*R*)-5-en-spirostane-2*α*,3*β*-diol 3-*O*-*α*-*L*-rhamnopyr-anosyl-(1→ 2)-[*α*-*L*-rhamnopyranosyl-(1→4)]-*β*-D-glucopyranoside | Steroid |  | α-glucosidase inhibition (IC_50_ 14.01 µM) | [119] |

Streptozotocin (STZ), protein tyrosine phosphatase1B (PTP1B), high-fat diet (HFD), Peroxisome proliferator–activated receptor (PPAR), endoplasmic-reticulum (ER)

**References**

1. DeFilipps RA, Krupnick GA. The medicinal plants of Myanmar. PhytoKeys 2018, DOI: 10.3897/phytokeys.102.24380: 1-341

2. Awale S, Lin TZ, Than MM, Swe T, Saiki I, Kadota S. The healing art of traditional medicines in Myanmar. J Trad Med 2006; 23: 47-68

3. Nyunt T, Kyaw UAM, Nyunt UT, Win UM, Htay UH, Mint UW, Swe DT, Yee DKW, Zaw MW, Cho LL, Than DMM, Kyaw DZM. Medicinal plants of myanmar. In: medicine Mohdot ed; 2007

4. Thu ZM, Aye MM, Aung HT, Sein MM, Vidari G. A Review of Common Medicinal Plants in Chin State, Myanmar. Natural Product Communications 2018; 13: 1557-1567

5. Agarwal R, Sulaiman SA, Mohamed M. Open Label Clinical Trial to Study Adverse Effects and Tolerance to Dry Powder of the Aerial Part of Andrographis Paniculata in Patients Type 2 with Diabetes Mellitus. Malaysian Journal of Medical Sciences 2005; 12: 13-19

6. Prisilla DH, Balamurugan R, Shah HR. Antidiabetic activity of methanol extract of *Acorus calamus* in STZ induced diabetic rats. Asian Pacific Journal of Tropical Biomedicine 2012; 2: S941-S946

7. Ashraf R, Khan R, I A. Garlic (*Allium sativum*) supplementation with standard antidiabetic agent provides better diabetic control in type 2 diabetes patients. Pak J Pharm Sci 2011; 24: 565-570

8. Zhang CT, Ma YL, Zhao YX, Hong YQ, Cai SB, Pang MJ. Phenolic composition, antioxidant and pancreatic lipase inhibitory activities of Chinese sumac (*Rhus chinensis* Mill.) fruits extracted by different solvents and interaction between myricetin-3-*O*-rhamnoside and quercetin-3-*O*-rhamnoside. Int J Food Sci Tech 2018; 53: 1045-1053

9. Prakash V, Jaiswal N, Srivastava M. A Review on Medicinal Properties of *Centella Asiatica*. Asian Journal of Pharmaceutical and Clinical Research 2017; 10:

10. Anitha T, Balakumar C, Ilango KB, Jose CB, Vetrivel D. Antidiabetic activity of the aqueous extracts of *Foeniculum vulgare* on streptozotocin-induced diabetic rats. Int J Adv Pharm Biol Chem 2014; 3: 487-494

11. Laleye FOA, Mensah S, Assogbadjo AE, Ahissou H. Diversity, Knowledge, and Use of Plants in Traditional Treatment of Diabetes in the Republic of Benin. Ethnobotany Research and Applications 2015; 14: 231-257

12. Bhusal A, Jamarkattel N, Shrestha A, Lamsal NK, Shakya S, Rajbhandari S. Evaluation of antioxidative and antidiabetic activity of bark of holarrhena pubescens wall. J Clin Diagn Res 2014; 8: HC05-08

13. Bihani T. *Plumeria rubra* L.- A review on its ethnopharmacological, morphological, phytochemical, pharmacological and toxicological studies. J Ethnopharmacol 2020; 264: 113291

14. Qureshi SA, Nawaz A, Udani SK, Azmi B. Hypoglycaemic and Hypolipidemic Activities of *Rauwolfia serpentina* in Alloxan-Induced Diabetic Rats. International Journal of Pharmacology 2009; 5: 323-326

15. Bhatia A, Anand M, Singla R. Efficacy of In Vitro and Native *Tylophora Indica* Leaf Extract against Hyperglycemic Mice Induced with Alloxan through Oral Administration. International Journal of Chemical and Pharmaceutical Analysis 2015; 2: 88-92

16. Lee YM, Kim YS, Kim JH, Kim JS. Screening of Chinese Herbal Medicines with Inhibitory Effect on Aldose Reductase (V). Kor J Pharmacogn 2011; 42: 187-194

17. Sanjay Kumar K. Isolation of β-sitosterol and evaluation of antidiabetic activity of *Aristolochia indica* in alloxan-induced diabetic mice with a reference to in-vitro antioxidant activity. Journal of Medicinal Plants Research 2012; 6:

18. Kumar VK, Lalitha KG. In vitro study on α-amylase inhibitory activity of an Ayurvedic medicinal plant, *Anacyclus pyrethrum* DC root. Indian J Pharmacol 2014; 46: 350-351

19. Tyagi S, Mansoori MH, Singh NK, Shivhare MK, Bhardwaj P, Singh RK. Antidiabetic Effect of *Anacyclus pyrethrum* DC in Alloxan Induced Diabetic Rats. European Journal of Biological Sciences 2011; 3: 117-120

20. Nguyen MTT, Nguyen NT, Nguyen HX, Huynh TNN, Min BS. Screening of α-Glucosidase Inhibitory Activity of Vietnamese Medicinal Plants: Isolation of Active Principles from *Oroxylum indicum*. Natural Product Sciences 2012; 18: 47-51

21. Attia ES, Amer AH, Hasanein MA. The hypoglycemic and antioxidant activities of garden cress (*Lepidium sativum* L.) seed on alloxan-induced diabetic male rats. Nat Prod Res 2019; 33: 901-905

22. Chakrabarti R, Singh BA, Vn P, Vanchhawng LT, Thirumurugan K. Screening of nine herbal plants for in vitro α-amylase inhibition. Asian Journal of Pharmaceutical and Clinical Research 2014; 7: 84-89

23. Balekari U, Veeresham C. Insulinotropic activity of methanolic extract of *Mesua ferrea*. Journal of Basic & Applied Sciences 2015; 11: 410-417

24. J AB, Shihabudeen MS, Thirumurugan K. Screening of fifteen Indian ayurvedic plants for α-glucosidase inhibitory activity and enzyme kinetics. International Journal of Pharmacy and Pharmaceutical Sciences 2011; 3: 267-274

25. Chen CY, Yan ZY, Li SH, Chen C, Zuo CY. Effect of the Extract of *Valeriana jatamansi* on the Blood Lipid and Liver Function in Experimental Hyperlipidemia Rats. Chinese Journal of Experimental Traditional Medical Formulae 2012; 18: 154-157

26. Ali MY, Paul S, Tanvir EM, Hossen MS, Rumpa NN, Saha M, Bhoumik NC, Aminul Islam M, Hossain MS, Alam N, Gan SH, Khalil MI. Antihyperglycemic, Antidiabetic, and Antioxidant Effects of *Garcinia pedunculata* in Rats. Evidence-based complementary and alternative medicine : eCAM 2017; 2017: 2979760

27. Feng L, Zhai YY, Xu J, Yao WF, Cao YD, Cheng FF, Bao BH, Zhang L. A review on traditional uses, phytochemistry and pharmacology of *Eclipta prostrata* (L.) L. J Ethnopharmacol 2019; 245: 112109

28. Marianne, P DL, Sukandar EY, Kurniati NF, Nasution R. Antidiabetic Activity of Leaves Ethanol Extract *Chromolaena odorata* (L.) R.M. King on Induced Male Mice with Alloxan Monohydrate. Jurnal Natural 2014; 14: 1-4

29. Kumar M, Guleria S, Chawla P, Khan A, Modi VK, Kumar N, Kaushik R. Anti-obesity efficacy of the selected high altitude Himalayan herbs: in vitro studies. J Food Sci Technol 2020; 57: 3081-3090

30. Chunudom L, Thongsom M, Karim N, Rahman MA, Rana MN, Tangpong J. *Tithonia diversifolia* aqueous fraction plays a protective role against alloxan-induced diabetic mice via modulating GLUT2 expression. S Afr J Bot 2020; 133: 118-123

31. Rath D, Kar DM, Panigrahi SK, Maharana L. Antidiabetic effects of *Cuscuta reflexa* Roxb. in streptozotocin induced diabetic rats. J Ethnopharmacol 2016; 192: 442-449

32. Chawda HM, Mandavia DR, Parmar PH, Baxi SN, Tripathi CR. Hypolipidemic activity of a hydroalcoholic extract of *Cyperus scariosus* Linn. root in guinea pigs fed with a high cholesterol diet. Chin J Nat Medicines 2014; 12: 0819−0826

33. Karthik VP, Suresh P, David DC. In Vitro Hydrogen Peroxide Scavenging Activity and Alpha Amylase Inhibitory Activity of *Croton tiglium* extract. Research Journal of Pharmacy and Technology 2019; 12: 3045-3047

34. Mohan RS, Silambujanaki P, Chitra V, Raju D. A Review of Malaysian Medicinal Plants with Potential Antidiabetic Activity. Int J Res Pharmacol Pharmacotherap 2013; 2: 263-266

35. Nwanjo HU. Hypoglycemic and Hypolipidemic Effects of Aqueous Extracts of *Abrus precatorius* Linn Seeds in Streptozotocin-Induced Diabetic Wistar Rats. J Herbs, Spices Med Plants 2008; 14: 68-76

36. Goyal M. Traditional plants used for the treatment of diabetes mellitus in Sursagar constituency, Jodhpur, Rajasthan - An ethnomedicinal survey. J Ethnopharmacol 2015; 174: 364-368

37. Kumar R, Patel DK, Prasad SK, Sairam K, Hemalatha S. Antidiabetic activity of alcoholic root extract of *Caesalpinia digyna* in streptozotocin-nicotinamide induced diabetic rats. Asian Pacific Journal of Tropical Biomedicine 2012; 2: S934-S940

38. Qureshi JA, Memon Z, Mirza KM, Agha S, Saher F, Sunderjee NF. Antihyperglycemic and Antihyperlipidemic Activity of *Linum usitatissimum* and *Glycyrrhiza glabra* Extracts in Streptozotocin-Induced Diabetic Rats. Asian Journal of Research in Medical and Pharmaceutical Sciences 2018; 5: 1-10

39. Badole SL, Subhash, Bodhankar L. Antihyperglycemic Activity of *Pongamia pinnata* Stem Bark in Diabetic Mice. Pharm Biol 2009; 46: 900-905

40. Challa CS, Lokesh T, Devanna Nayakanti N. Anti-Diabetic and antimicrobial activity of *Pterocarpus santalinus* heart wood. Research Journal of Life Sciences, Bioinformatics, Pharmaceutical and Chemical Sciences 2019; 5: 1190-1199

41. Verma N, Usman K, Patel N, Jain A, Dhakre S, Swaroop A, Bagchi M, Kumar P, Preuss HG, Bagchi D. A multicenter clinical study to determine the efficacy of a novel fenugreek seed (*Trigonella foenum*-*graecum*) extract (Fenfuro) in patients with type 2 diabetes. Food Nutr Res 2016; 60: 32382

42. Neelakantan N, Narayanan M, Souza RJd, Dam RMv. Effect of fenugreek (*Trigonella foenum*-*graecum* L.) intake on glycemia: a meta-analysis of clinical trials. Nutrition Journal 2014; 13:

43. Zheng T, Shu GW, Yang ZZ, Mo SS, Zhao Y, Mei ZN. Antidiabetic effect of total saponins from *Entada phaseoloides* (L.) Merr. in type 2 diabetic rats. J Ethnopharmacol 2012; 139: 814-821

44. Manosroi J, Moses ZZ, Manosroi W, Manosroi A. Hypoglycemic activity of Thai medicinal plants selected from the Thai/Lanna Medicinal Recipe Database MANOSROI II. J Ethnopharmacol 2011; 138: 92-98

45. Somtimuang C, Olatunji OJ, Ovatlarnporn C. Evaluation of In Vitro α-Amylase and α-Glucosidase Inhibitory Potentials of 14 Medicinal Plants Constituted in Thai Folk Antidiabetic Formularies. Chem Biodivers 2018; 15: e1800025

46. Chhetri DR, Parajuli P, Subba GC. Antidiabetic plants used by Sikkim and Darjeeling Himalayan tribes, India. J Ethnopharmacol 2005; 99: 199-202

47. Huang XL, Wan D, Shu J, Fang LZ, Zou DZ, Zhang SH. α-Glucosidase Inhibitory Activity-guided Identification of Compounds from *Clerodendrum bungei* Steud by HPLC-ESI- QTOF-MS/MS. Digital Chinese Medicine 2019; 2: 41-49

48. Ekoh SN, Akubugwo EI, Ude3 VC, Edwin N. Anti-hyperglycemic and anti-hyperlipidemic effect of spices (*Thymus vulgaris*, *Murraya koenigii*, *Ocimum gratissimum* and *Piper guineense*) in alloxan-induced diabetic rats. International Journal of Biosciences (IJB) 2014, DOI: 10.12692/ijb/4.2.179-187: 179-187

49. Nishina A, Itagaki M, Sato D, Kimura H, Hirai Y, Phay N, Makishima M. The Rosiglitazone-Like Effects of Vitexilactone, a Constituent from *Vitex trifolia* L. in 3T3-L1 Preadipocytes. Molecules 2017; 22:

50. Gogoi B, Kakoti BB, Borah S, Borah NS. Antihyperglycemic and in vivo antioxidative activity evaluation of *Cinnamomum bejolghota* (Buch.-Ham.) in streptozotocin induced diabetic rats: an ethnomedicinal plant in Assam. Asian Pacific Journal of Tropical Medicine 2014; 7: S427-S434

51. Bisht S, Sisodia SS. Assessment of antidiabetic potential of *Cinnamomum tamala* leaves extract in streptozotocin induced diabetic rats. Indian Journal of Pharmacology 2011; 43: 582-585

52. Chakraborty U, Das H. Antidiabetic and Antioxidant Activities of *Cinnamomum tamala* Leaf Extracts in STZ-Treated Diabetic Rats. Global Journal of Biotechnology & Biochemistry 2010; 5: 12-18

53. Chakraborty R, Mandal V. In vitro Hypoglycemic and Antioxidant Activities of *Litsea cubeba* (Lour.) Pers. fruits, Traditionally used to Cure Diabetes in Darjeeling Hills (India). Pharmacognosy Journal 2018; 10: s119-s128

54. Biswas A, Chatterjee S, Chowdhury R, Sen S, Sarkar DIPAK, Chatterjee M, Das J. Antidiabetic effect of seeds of *Strychnos potatorum* Linn. in a streptozotocin-induced model of diabetes. Acta Pol Pharm 2012; 69: 939-943

55. Judy WV, Hari SP, Stogsdill WW, Judy JS, Naguib YMA, Passwater R. Antidiabetic activity of a standardized extract (Glucosol™) from *Lagerstroemia speciosa* leaves in Type II diabetics. J Ethnopharmacol 2003; 87: 115-117

56. Kumar AS, Kavimani S, Jayaveera KN. Anti-diabetic potential of methanol extract of *Grewia tiliaefolia* stem bark in streptozotocin-induced diabetic rats Asian Pacific Journal of Tropical Biomedicine. Asian Pacific Journal of Tropical Biomedicine 2012, DOI: 1-6

57. Elbashir SMI, Devkota HP, Wada M, Kishimoto N, Moriuchi M, Shuto T, Misumi S, Kai H, Watanabe T. Free radical scavenging, α-glucosidase inhibitory and lipase inhibitory activities of eighteen Sudanese medicinal plants. BMC complementary and alternative medicine 2018; 18: 282

58. Kumari DJ. Hypoglycemic effect of *Moringa oleifera* and *Azadirachta indica* in type-2 diabetes. Bioscan 2010; 5: 211-214

59. Karunasree V, Veeresham C, Sambasiva Rao KRS, Asres K. Evaluation of the anti diabetic activity of column fractions obtained from the bark extract of *Soymida febrifuga* A. Juss. Pharmacognosy Journal 2012; 4: 37-43

60. Piero NM. In Vivo Antidiabetic Activity and Safety In Rats of *Cissampelos pareira* Traditionally Used In The Management of Diabetes Mellitus In Embu County, Kenya. Journal of Drug Metabolism & Toxicology 2015; 06:

61. Shirwaikar A, Rajendran K, Punitha IS. Antidiabetic activity of alcoholic stem extract of *Coscinium fenestratum* in streptozotocin-nicotinamide induced type 2 diabetic rats. J Ethnopharmacol 2005; 97: 369-374

62. Abu MN, Samat S, Kamarapani N, Nor Hussein F, Wan Ismail WI, Hassan HF. *Tinospora crispa* Ameliorates Insulin Resistance Induced by High Fat Diet in Wistar Rats. Evidence-based complementary and alternative medicine : eCAM 2015; 2015: 985042

63. Banerjee A, Maji B, Mukherjee S, Chaudhuri K, Seal T. In Vitro Antidiabetic and Anti-oxidant Activities of Methanol Extract of *Tinospora Sinensis*. Journal of Applied Biology & Biotechnology 2017; 5: 61-67

64. Sahu SK, Das D, Tripathy NK, Dinda SC, Sundeep Kumar HK. Evaluation of hypoglycemic activity of *Mollugo pentaphylla* and *Glinus oppositifolius* (L). Rasayan Journal of Chemistry 2012; 5: 57-62

65. Prashar Y, Patel NJ. An in vitro approach to evaluate the anti-adipogenic effect of *Myrica nagi* Thunb. fruit extract on 3T3-L1 adipocyte cell line. Obesity Medicine 2020; 18:

66. Kim MS, Ahn SM, Jung IC, Kwon GS, Sohn HY. Screening of α-Amylase and α-Glucosidase Inhibitor from Nepalese Plant Extracts. Kor J Microbiol Biotechnol 2010; 38: 183–189

67. Somani RS, Singhai AK. Hypoglycaemic and antidiabetic activities of seeds of *Myristica fragrans* in normoglycaemic and alloxan-induced diabetic rats. Asian J Exp Sci 2008; 22: 95-102

68. Salehi P, Asghari B, Esmaeili MA, Dehghan H, Ghazi I. α-Glucosidase and α-amylase inhibitory effect and antioxidant activity of ten plant extracts traditionally used in Iran for diabetes. Journal of Medicinal Plants Research 2013; 7: 257-266

69. Chaudhry ZR, Chaudhry SR, Naseer A, Chaudhry FR. Effect of *Syzygium aromaticum* (clove) extract on blood glucose level in streptozotocin induced diabetic rats. Pakistan Armed Forces Medical Journal 2013; 63: 323-328

70. Islam D, Huque A, Sheuly, Mohanta LC, Das SK, Sultana A, Lipy EP, Prodhan UK. Hypoglycemic and hypolipidemic effects of *Nelumbo nucifera* flower in Long-Evans rats. Journal of Herbmed Pharmacology 2018; 7: 148-154

71. Khan MH, Yadava PS. Antidiabetic plants used in Thoubal district of Manipur, Northeast India. Indian Journal of Traditional Knowledge 2010; 9: 510-514

72. Vijayakumar RS, Surya, D., Senthilkumar, R., & Nalini, N. Hypolipidemic effect of black pepper (*Piper nigrum* Linn.) in rats fed high fat diet. Journal of clinical biochemistry and nutrition 2002; 32: 31-42

73. Phumthum M, Balslev H. Thai Ethnomedicinal Plants Used for Diabetes Treatment. OBM Integrative and Complementary Medicine 2018; 3: 1-1

74. Kumar S, Patial V, Soni S, Sharma S, Pratap K, Kumar D, Padwad Y. *Picrorhiza kurroa* Enhances beta-Cell Mass Proliferation and Insulin Secretion in Streptozotocin Evoked β-Cell Damage in Rats. Front Pharmacol 2017; 8: 537

75. Zarmouh MM, Subramaniyam K, Viswanathan S, Kumar PG. Cause and effect of *Plumbago zeylanica* root extract on blood glucose and hepatic enzymes in experimental diabetic rats. African Journal of Microbiology Research 2010; 4: 2674-2677

76. Ramya SS, Vijayanand N, Rathinavel S. Antidiabetic activity of *Cynodon dactylon* (l.) pers. extracts in alloxan induced rats. Int J Pharm Pharm Sci 2014; 6: 348-352

77. Reddy JK, Sheetal P, Menda R, Aparna N. Anti diabetic activity of ethanolic extract of *Aconitum ferox* in alloxan induced diseased rats. International Journal of Pharmacy and Biological Sciences 2016; 6: 125-134

78. Tag H, Kalita P, Dwivedi P, Das AK, Namsa ND. Herbal medicines used in the treatment of diabetes mellitus in Arunachal Himalaya, northeast, India. J Ethnopharmacol 2012; 141: 786-795

79. Najmi A, Nasiruddin M, Khan RA, Haque SF. Effect of *Nigella sativa* oil on various clinical and biochemical parameters of insulin resistance syndrome. International journal of diabetes in developing countries 2008; 28: 11-14

80. Badar A, Kaatabi H, Bamosa A, Al-Elq A, Abou-Hozaifa B, Lebda F, Alkhadra A, Al-Almaie S. Effect of *Nigella sativa* supplementation over a one-year period on lipid levels, blood pressure and heart rate in type-2 diabetic patients receiving oral hypoglycemic agents: nonrandomized clinical trial. Ann Saudi Med 2017; 37: 56-63

81. Junejo JA, Rudrapal M, Zaman K. Antidiabetic activity of *Carallia brachiata* Lour. leaves hydro-alcoholic extract (HAE) with antioxidant potential in diabetic rats. Indian Journal of Natural Products and Resources 2020; 11: 18-29

82. Neamsuvan O, Madeebing N, Mah L, Lateh W. A survey of medicinal plants for diabetes treating from Chana and Nathawee district, Songkhla province, Thailand. J Ethnopharmacol 2015; 174: 82-90

83. Kulkarni CR, Joglekar MM, Patil SB, Arvindekar AU. Antihyperglycemic and antihyperlipidemic effect of *Santalum album* in streptozotocin induced diabetic rats. Pharm Biol 2012; 50: 360-365

84. Morikawa T, Xie YY, Asao Y, Okamoto M, Yamashita C, Muraoka O, Matsuda H, Pongpiriyadacha Y, Yuan D, Yoshikawa M. Oleanane-type triterpene oligoglycosides with pancreatic lipase inhibitory activity from the pericarps of *Sapindus rarak*. Phytochemistry 2009; 70: 1166-1172

85. Ganu G, Jadhav S. In Vitro Antioxidant and In Vivo Antihyperglycemic Potential of *Mimusops elengi* L. in Alloxan-Induced Diabetes in Mice. Journal of Complementary and Integrative Medicine 2010; 7:

86. Ablat A, Halabi MF, Mohamad J, Hasnan MH, Hazni H, Teh SH, Shilpi JA, Mohamed Z, Awang K. Antidiabetic effects of *Brucea javanica* seeds in type 2 diabetic rats. BMC complementary and alternative medicine 2017; 17: 94

87. Sen S, Chakraborty R, De B, Devanna N. An ethnobotanical survey of medicinal plants used by ethnic people in West and South district of Tripura, India. Journal of Forestry Research 2011; 22: 417-426

88. Kumar S, Saini M, Kumar V, Prakash O, Arya R, Rana M, Kumar D. Traditional medicinal plants curing diabetes: A promise for today and tomorrow. Asian J Tradit Med 2012; 7: 178-188

89. Chatsumpun N, Sritularak B, Likhitwitayawuid K. New Biflavonoids with α-Glucosidase and Pancreatic Lipase Inhibitory Activities from *Boesenbergia rotunda*. Molecules 2017; 22:

90. Prasannarong M, Saengsirisuwan V, Piyachaturawat P, Suksamrarn A. Improvements of insulin resistance in ovariectomized rats by a novel phytoestrogen from *Curcuma comosa* Roxb. BMC complementary and alternative medicine 2012; 12:

91. Sudatri NW, Warasiti N, Suartini NM, Bidura NI. Anti-diabetic and anti-cholesterol activity of *Kaempferia galanga* L. herbal medicine rhizome in albino rats. International Journal of Fauna and Biological Studies 2019; 6: 13-17

92. Mahluji S, Attari VE, Mobasseri M, Payahoo L, Ostadrahimi A, Golzari SE. Effects of ginger (*Zingiber officinale*) on plasma glucose level, HbA1c and insulin sensitivity in type 2 diabetic patients. Int J Food Sci Nutr 2013; 64: 682-686

93. Brindis F, Rodriguez R, Bye R, Gonzalez-Andrade M, Mata R. (*Z*)-3-butylidenephthalide from *Ligusticum porteri* , an α-glucosidase inhibitor. J Nat Prod 2011; 74: 314-320

94. Abima Shazhni JR, Renu A, Vijayaraghavan P. Insights of antidiabetic, anti-inflammatory and hepatoprotective properties of antimicrobial secondary metabolites of corm extract from *Caladium x hortulanum*. Saudi journal of biological sciences 2018; 25: 1755-1761

95. Goverdhan P, Rani MS, K.Thirupathi, Rani S, Sathesh S, Kumar BR, Mohan GK. Hypoglycemic and Antihyperglycemic Effect of *Aristolochia Indica* Normal and Alloxan Induced Diabetic Rats. Pharmacologyonline 2008; 1: 20-29

96. Das A, Chhetry TK. Evaluation of Anti-Diabetic Activity of *Mahonia nepalensis* in STZ Induced Rat Model. International Journal of Pharmacognosy and Phytochemical Research 2016; 8: 1104-1110

97. Ibrahim RB, Akolade JO, Aladodo RA, Okereke OE, Akande SA. Glucose and Lipid Lowering Potentials of *Heliotropium indicum* L. Leaves in Alloxan-Induced Hyperglycaemic Rats. Not Sci Biol 2016; 8: 414 -421

98. Mahamat Nour Adam S. In vitro anti-hyperglycaemic effect of glucocapparin isolated from the seeds of *Boscia senegalensis* (Pers.) Lam. ex Poiret parasuis. African Journal of Biotechnology 2012; 11:

99. Lee DY, Kim HW, Yang H, Sung SH. Hydrolyzable tannins from the fruits of *Terminalia chebula* Retz and their α-glucosidase inhibitory activities. Phytochemistry 2017; 137: 109-116

100. Nagappa AN, Thakurdesai PA, Venkat Rao N, Singh J. Antidiabetic activity of *Terminalia catappa* Linn fruits. J Ethnopharmacol 2003; 88: 45-50

101. Saratale GD, Saratale RG, Benelli G, Kumar G, Pugazhendhi A, Kim D-S, Shin H-S. Anti-diabetic Potential of Silver Nanoparticles Synthesized with *Argyreia nervosa* Leaf Extract High Synergistic Antibacterial Activity with Standard Antibiotics Against Foodborne Bacteria. Journal of Cluster Science 2017; 28: 1709-1727

102. Uchegbu NN, Ishiwu CN. Germinated Pigeon Pea (*Cajanus cajan*): a novel diet for lowering oxidative stress and hyperglycemia. Food science & nutrition 2016; 4: 772-777

103. Yi YR, Lu W, Jie X, Xiang L, Shan L, Xiang QS, Jie HY, Ling SX. Treatment of type 2 diabetes mellitus via reversing insulin resistance and regulating lipid homeostasis in vitro and in vivo using cajanonic acid A. Int J Mol Med 2018; 42: 2329-2342

104. Hariharan R, Venkataraman S, Sunitha P, Rajalakshmi S, Samal K, Routray B, Jayakumar R, Baiju K, Satyavati G, Muthuswamy V. Efficacy of vijayasar (*Pterocarpus marsupium*) in the treatment of newly diagnosed patients with type 2 diabetes mellitus a flexible dose double-blind Diabetologia Croatica 2005; 34: 13–20

105. Irshad N, Akhtar MS, Bashir S, Hussain A, Shafiq M, Iqbal J, Malik A. Hypoglycaemic effects of Methanolic extract of *Canscora decussata* (Schuh) whole-plant in normal and alloxan-induced diabetic rabbits. Pak J Pharm Sci 2015; 28: 167-174

106. Kumari ST, Sakthidevi G, Muthukumaraswamy S, Mohan VR. Hypoglycemic and Hypolipidemic Effects of Ethanol Extract of *Canscora Perfoiata* Lam.(Gentianaceae) Whole Plant in Alloxan Induced Diabetic Rats. International Journal of Pharmacy and Pharmaceutical Sciences 2013; 5: 518-522

107. Purintrapiban J, Keawpradub N, Kansenalak S, Chittrakarn S, Janchawee B, Sawangjaroen K. Study on glucose transport in muscle cells by extracts from *Mitragyna speciosa* (Korth) and mitragynine. Nat Prod Res 2011; 25: 1379-1387

108. Zhao G, Li X, Chen W, Xi Z, Sun L. Three new sesquiterpenes from *Tithonia diversifolia* and their anti-hyperglycemic activity. Fitoterapia 2012; 83 1590–1597

109. Lin HR. Sesquiterpene lactones from *Tithonia diversifolia* act as peroxisome proliferator-activated receptor agonists. Bioorganic & medicinal chemistry letters 2012; 22: 2954-2958

110. Dai Y, Chen SR, Chai L, Zhao J, Wang Y, Wang Y. Overview of pharmacological activities of Andrographis paniculata and its major compound andrographolide. Crit Rev Food Sci Nutr 2019; 59: S17-S29

111. Mukherjee A, Sengupta S. Characterization of nimbidiol as a potent intestinal disaccharidase and glucoamylase inhibitor present in *Azadirachta indica* (neem) useful for the treatment of diabetes. J Enzyme Inhib Med Chem 2013; 28: 900-910

112. Perez-Gutierrez RM, Damian-Guzman M. Meliacinolin: A Potent α-Glucosidase and α-Amylase Inhibitor Isolated from *Azadirachta indica* Leaves and in Vivo Antidiabetic Property in Streptozotocin-Nicotinamide-Induced Type 2 Diabetes in Mice. Biological and pharmaceutical bulletin 2012; 35: 1516-1524

113. Yonemoto R, Shimada M, Gunawan-Puteri MD, Kato E, Kawabata J. α-Amylase inhibitory triterpene from *Abrus precatorius* leaves. J Agric Food Chem 2014; 62: 8411-8414

114. Zhang SN, Song HZ, Ma RJ, Liang CQ, Wang HS, Tan QG. Potential anti-diabetic isoprenoids and a long-chain delta-lactone from frangipani (*Plumeria rubra*). Fitoterapia 2020; 146: 104684

115. Badole SL, Bodhankar SL. Antidiabetic activity of cycloart-23-ene-3β, 25-diol (B2) isolated from *Pongamia pinnata* (L. Pierre) in streptozotocin–nicotinamide induced diabetic mice. Eur J Pharmacol 2010; 632: 103–109

116. Xiong H, Zhang S, Zhao Z, Zhao P, Chen L, Mei Z. Antidiabetic activities of entagenic acid in type 2 diabetic db/db mice and L6 myotubes via AMPK/GLUT4 pathway. J Ethnopharmacol 2018; 211: 366-374

117. Le DD, Nguyen DH, Ma ES, Lee JH, Min BS, Choi JS, Woo MH. PTP1B Inhibitory and Anti-inflammatory Properties of Constituents from *Eclipta prostrata* L. Biol Pharm Bull 2021; 44: 298–304

118. Hou W, Li Y, Zhang Q, Wei X, Peng A, Chen L, Wei Y. Triterpene acids isolated from *Lagerstroemia speciosa* leaves as α-glucosidase inhibitors. Phytotherapy research : PTR 2009; 23: 614-618

119. Zhang H, Xu J, Wang M, Xia X, Dai R, Zhao Y. Steroidal saponins and sapogenins from fenugreek and their inhibitory activity against alpha-glucosidase. Steroids 2020; 161: 108690

120. Swapna K, Sathibabu Uddandrao VV, Parim B, Ravindarnaik R, Suresh P, Ponnusamy P, Balakrishnan S, Vadivukkarasi S, Harishankar N, Reddy KP, Nivedha PR, Saravanan G. Effects of asiatic acid, an active constituent in *Centella asiatica* (L.): restorative perspectives of streptozotocin-nicotinamide induced changes on lipid profile and lipid metabolic enzymes in diabetic rats. Comparative Clinical Pathology 2019; 28: 1321-1329

121. Wang X, Lu Q, Yu D-S, Chen Y-P, Shang J, Zhang L-Y, Sun H-B, Liu J. Asiatic acid mitigates hyperglycemia and reduces islet fibrosis in Goto-Kakizaki rat, a spontaneous type 2 diabetic animal model. Chin J Nat Medicines 2015; 13: 529-534

122. Lahrita L, Moriai K, Iwata R, Itoh K, Kato E. Quassinoids in *Brucea javanica* are potent stimulators of lipolysis in adipocytes. Fitoterapia 2019; 137: 104250

123. NoorShahida A, Wong TW, Choo CY. Hypoglycemic effect of quassinoids from *Brucea javanica* (L.) Merr (Simaroubaceae) seeds. J Ethnopharmacol 2009; 124: 586-591

124. Payum T. Distribution, Ethnobotany, Pharmacognosy and Phytoconstituents of *Coptis teeta* Wall.: A Highly Valued and Threatened Medicinal Plant of Eastern Himalayas. Pharmacognosy Journal 2017; 9: s28-s34

125. Lan J, Zhao Y, Dong F, Yan Z, Zheng W, Fan J, Sun G. Meta-analysis of the effect and safety of berberine in the treatment of type 2 diabetes mellitus, hyperlipemia and hypertension. J Ethnopharmacol 2015; 161: 69-81

126. Choi JS, Kim JH, Ali MY, Min BS, Kim GD, Jung HA. *Coptis chinensis* alkaloids exert anti-adipogenic activity on 3T3-L1 adipocytes by downregulating C/EBP-α and PPAR-γ. Fitoterapia 2014; 98: 199-208

127. Sama V, Nadipelli M, Yenumula P, Bommineni MR, Mullangi R. Effect of piperine on antihyperglycemic activity and pharmacokinetic profile of nateglinide. Arzneimittelforschung 2012; 62: 384-388

128. Wu XL, Deng MZ, Gao ZJ, Dang YY, Li YC, Li CW. Neferine alleviates memory and cognitive dysfunction in diabetic mice through modulation of the NLRP3 inflammasome pathway and alleviation of endoplasmic-reticulum stress. Int Immunopharmacol 2020; 84: 106559

129. Yang X, Huang M, Yang J, Wang J, Zheng S, Ma X, Cai J, Deng S, Shu G, Yang G. Activity of Isoliensinine in Improving the Symptoms of Type 2 Diabetic Mice via Activation of AMP-Activated Kinase and Regulation of PPARgamma. J Agric Food Chem 2017; 65: 7168-7178

130. Yuan T, Nahar P, Sharma M, Liu K, Slitt A, Aisa HA, Seeram NP. Indazole-type alkaloids from *Nigella sativa* seeds exhibit antihyperglycemic effects via AMPK activation in vitro. J Nat Prod 2014; 77: 2316-2320

131. Parveen A, Farooq MA, Kyunn WW. A New Oleanane Type Saponin from the Aerial Parts of *Nigella sativa* with Anti-Oxidant and Anti-Diabetic Potential. Molecules 2020; 25:

132. Kuroda M, Mimaki Y, Honda S, Tanaka H, Yokota S, Mae T. Phenolics from Glycyrrhiza glabra roots and their PPAR-gamma ligand-binding activity. Bioorganic & medicinal chemistry 2010; 18: 962-970

133. Nuankaew W, Heemman A, Wattanapiromsakul C, Shim JH, Kim NW, Yasmin T, Jeong SY, Nam YH, Hong BN, Dej-Adisai S, Kang TH. Anti-insulin resistance effect of constituents from *Senna siamea* on zebrafish model, its molecular docking, and structure-activity relationships. J Nat Med 2021, DOI: 10.1007/s11418-021-01490-5:

134. Wang XY, Zhu BR, Jia Q, Li YM, Wang T, Wang HY. Cinnamtannin D1 Protects Pancreatic beta-Cells from Glucolipotoxicity-Induced Apoptosis by Enhancement of Autophagy In Vitro and In Vivo. J Agric Food Chem 2020; 68: 12617-12630

135. Yang S, Na M, Jang JP, Kim KA, Kim BY, Sung NJ, Oh WK, Ahn JS. Inhibition of Protein Tyrosine Phosphatase 1Bby Lignans from *Myristica fragrans*. Phytother Res 2006; 20: 680–682

136. Nguyen PH, Le TV, Kang HW, Chae J, Kim SK, Kwon KI, Seo DB, Lee SJ, Oh WK. AMP-activated protein kinase (AMPK) activators from *Myristica fragrans* (nutmeg) and their anti-obesity effect. Bioorganic & medicinal chemistry letters 2010; 20: 4128-4131

137. Han KL, Choi JS, Lee JY, Song J, Joe MK, Jung MH, Hwang JK. Therapeutic potential of peroxisome proliferators-activated receptor-α-/γ dual agonist with alleviation of endoplasmic reticulum stress for the treatment of diabetes. Diabetes 2008; 57: 737-745

138. Wang F, Zhang L, Li BJ, Liang QM, Chen AL, Fang ZF, Wang SM. Cysestermerol A, a rare stilbene sestermer with significant hypoglycemic activities from *Cynodon dactylon*. Nat Prod Res 2019, DOI: 10.1080/14786419.2019.1689501: 1-7

139. Kumar R, Patel DK, Prasad SK, Laloo D, Krishnamurthy S, Hemalatha S. Type 2 antidiabetic activity of bergenin from the roots of *Caesalpinia digyna* Rottler. Fitoterapia 2012; 83: 395-401

140. Lee SP, Kuo FY, Cheng JT, Wu MC. GLP-1 mediates the modulating effect of thymoquinone on feeding behaviors in diabetic rats. Diabetes Metab Syndr Obes 2019; 12: 873-881

141. Tiruppur Venkatachallam SK, Pattekhan H, Divakar S, Kadimi US. Chemical composition of *Nigella sativa* L. seed extracts obtained by supercritical carbon dioxide. J Food Sci Technol 2010; 47: 598-605

142. Sunil C, Duraipandiyan V, Agastian P, Ignacimuthu S. Antidiabetic effect of plumbagin isolated from *Plumbago zeylanica* L. root and its effect on GLUT4 translocation in streptozotocin-induced diabetic rats. Food and chemical toxicology : an international journal published for the British Industrial Biological Research Association 2012; 50: 4356-4363

143. Bharadwaja S, Issac PK, Cleta J, Jeganathan R, Chandrakumar SS, Sundaresan S. An in vitro mechanistic approach towards understanding the distinct pathways regulating insulin resistance and adipogenesis by apocynin. J Biosci (Bangalore) 2021; 46:

144. Wang Y, Tao J, Jiang M, Yao Y. Apocynin ameliorates diabetic retinopathy in rats: Involvement of TLR4/NF-kappaB signaling pathway. Int Immunopharmacol 2019; 73: 49-56

145. Antony PJ, Gandhi GR, Stalin A, Balakrishna K, Toppo E, Sivasankaran K, Ignacimuthu S, Al-Dhabi NA. Myoinositol ameliorates high-fat diet and streptozotocin-induced diabetes in rats through promoting insulin receptor signaling. Biomedicine & pharmacotherapy = Biomedecine & pharmacotherapie 2017; 88: 1098-1113

146. Haeri MR, Limaki HK, White CJ, White KN. Non-insulin dependent anti-diabetic activity of (2*S*, 3*R*, 4*S*) 4-hydroxyisoleucine of fenugreek (*Trigonella foenum graecum*) in streptozotocin-induced type I diabetic rats. Phytomedicine 2012; 19: 571-574
